# Complex Interplay between FleQ, Cyclic Diguanylate and Multiple σ Factors Coordinately Regulates Flagellar Motility and Biofilm Development in *Pseudomonas putida*

**DOI:** 10.1371/journal.pone.0163142

**Published:** 2016-09-16

**Authors:** Alicia Jiménez-Fernández, Aroa López-Sánchez, Lorena Jiménez-Díaz, Blanca Navarrete, Patricia Calero, Ana Isabel Platero, Fernando Govantes

**Affiliations:** Centro Andaluz de Biología del Desarrollo, Universidad Pablo de Olavide/Consejo Superior de Investigaciones Científicas/Junta de Andalucía, Sevilla, Spain; and Departamento de Biología Molecular e Ingeniería Bioquímica, Universidad Pablo de Olavide, Carretera de Utrera, Km. 1, 41013, Sevilla, Spain; Martin-Luther-Universitat Halle-Wittenberg, GERMANY

## Abstract

Most bacteria alternate between a free living planktonic lifestyle and the formation of structured surface-associated communities named biofilms. The transition between these two lifestyles requires a precise and timely regulation of the factors involved in each of the stages that has been likened to a developmental process. Here we characterize the involvement of the transcriptional regulator FleQ and the second messenger cyclic diguanylate in the coordinate regulation of multiple functions related to motility and surface colonization in *Pseudomonas putida*. Disruption of *fleQ* caused strong defects in flagellar motility, biofilm formation and surface attachment, and the ability of this mutation to suppress multiple biofilm-related phenotypes associated to cyclic diguanylate overproduction suggests that FleQ mediates cyclic diguanylate signaling critical to biofilm growth. We have constructed a library containing 94 promoters potentially involved in motility and biofilm development fused to *gfp* and *lacZ*, screened this library for FleQ and cyclic diguanylate regulation, and assessed the involvement of alternative σ factors σ^N^ and FliA in the transcription of FleQ-regulated promoters. Our results suggest a dual mode of action for FleQ. Low cyclic diguanylate levels favor FleQ interaction with σ^N^-dependent promoters to activate the flagellar cascade, encompassing the flagellar cluster and additional genes involved in cyclic diguanylate metabolism, signal transduction and gene regulation. On the other hand, characterization of the FleQ-regulated σ^N^- and FliA-independent P*lapA* and P*bcsD* promoters revealed two disparate regulatory mechanisms leading to a similar outcome: the synthesis of biofilm matrix components in response to increased cyclic diguanylate levels.

## Introduction

The alternation between a free-swimming planktonic lifestyle and the formation of structured highly cooperative polymer-encased sessile communities, named biofilms, is a common feature of bacterial life in the environment [1]. Biofilm formation is a form of coordinated collective behavior that has been regarded as an evolutionary precursor of developmental processes. Biofilm development proceeds through stages of adhesion, proliferation and microcolony formation and maturation, and is terminated by programmed biofilm dispersal [2,3]. Transition between the planktonic and biofilm lifestyles and through the different stages of biofilm development requires the timely production of different factors in response to environmental and physiological signals, involving a variety of signal transduction and regulatory pathways to connect such cues to the adequate physiological responses [3].

The nucleotide cyclic diguanylate (c-di-GMP) is a second messenger that is ubiquitously used in bacteria for signaling the transition between the planktonic and sessile lifestyles. c-di-GMP is synthesized from GTP by diguanylate cyclase (DGC) activities, associated to proteins with GGDEF domains, and degraded by specific phosphodiesterase (PDE) activities, associated to proteins with EAL or HD-GYP domains. Bacterial genomes often encode multiple proteins containing one or more of these domains, implying that the c-di-GMP levels are likely regulated in a complex fashion. Changes in c-di-GMP concentration are sensed by effectors, which in turn regulate a variety of processes, generally related to motility, biofilm development or virulence, acting at the transcriptional, translational or posttranslational levels. The biology of c-di-GMP signaling has been extensively reviewed in recent years [4–8].

FleQ has long been known as an enhancer-binding protein belonging to the NtrC/NifA family of σ^N^-dependent promoter activators, and as the master regulator of flagellar biogenesis in *Pseudomonas aeruginosa* and other bacteria [9–11]. In *P*. *aeruginosa*, flagellar promoters are regulated by a four-tiered cascade. Class I promoters drive the expression of the two major regulatory elements, the transcriptional activator FleQ and the alternative σ factor FliA [9, 10, 12]. FleQ directly activates a set of σ^N^-dependent Class II promoters, and indirectly activates Class III promoters, which are also σ^N^-dependent and activated by Class II gene products FleSR, and Class IV promoters, which are transcribed by FliA-loaded RNA polymerase. FleQ activation of several flagellar promoters is antagonized by c-di-GMP [13, 14], likely through high-affinity interaction with the AAA+ ATPase domain of FleQ [15, 16]. FleQ also represses transcription of σ^N^-independent promoters driving the synthesis of biofilm matrix components, and repression is released by interaction of FleQ with c-di-GMP [13, 17, 18]. In addition, FleQ positively regulates some of these operons at high c-di-GMP concentrations [17, 18]. The auxiliary protein FleN forms a complex with FleQ to antagonize its activity and enhance the action of c-di-GMP on both FleQ-activated [15, 19, 20] and repressed promoters [13, 17].

*Pseudomonas putida* is a well-characterized Gram-negative soil and rhizosphere bacterium. Due to its metabolic versatility, *P*. *putida* is a model organism for biodegradation of organic toxicants, and a key asset in bioremediation [21]. The ability to form biofilms on both biotic and abiotic surfaces is a key to *P*. *putida* survival in its natural environment, and several factors relevant to biofilm development in *P*. *putida* have been identified [22]. The high molecular weight adhesin proteins LapA and LapF are important determinants for cell-surface and cell-cell interactions in early and mature biofilms, respectively [23]. Flagella have been shown to contribute to initial surface attachment and the transition from microcolonies to the mature biofilm [22]. The extracellular matrix of *P*. *putida* biofilms contains extracellular DNA [24] and a mixture of exopolysaccharide (EPS). Four gene clusters involved in EPS synthesis and export are present in *P*. *putida* and the contribution of different types of EPS to the extracellular biofilm matrix and biofilm stability has been determined [25–28]. In addition, *P*. *putida* biofilms undergo rapid dispersal in response to nutritional stress [29]. Studies performed in *P*. *putida* and the related species *Pseudomonas fluorescens* have determined that dispersal is effected by proteolytic cleavage of LapA by the periplasmic protease LapG [6, 30, 31].

Despite the growing understanding of the structural and mechanistic details of the biofilm cycle, little is known of the signals and regulatory mechanisms governing biofilm development in *P*. *putida*. A role for c-di-GMP as a key regulator of biofilm formation is inferred from the aggregative hyper-biofilm forming phenotype obtained by overexpression of enzymes with diguanylate cyclase (DGC) activity [32, 33]. Although the *P*. *putida* KT2440 genome bears 43 genes encoding GGDEF, EAL or HD-GYP domain proteins [34, 35], only the putative DGC MorA and the PDE BifA have been shown to be unequivocally involved in biofilm development [36, 37]. Synthesis of the adhesin LapF, involved in biofilm maturation, is dependent on the stationary phase sigma factor σ^S^ [38], and a role of c-di-GMP and FleQ in the expression of *lapA* and the cellulose synthesis *bcs* operon was recently described [21, 39]. In addition, starvation-induced biofilm dispersal is triggered by a decrease in the intracellular c-di-GMP concentration effected by BifA [37]. Such decrease releases the inhibition of the protease activity of LapG by the membrane-bound c-di-GMP effector LapD, thus allowing LapA proteolysis [6, 30, 31].

Recently, we undertook the isolation of biofilm-deficient mutants of *P putida* KT2442 [40]. Five of our biofilm-deficient mutants were found to bear insertions in *fleQ*. Here we describe the phenotypic characterization of a null *fleQ* mutant and the impact of FleQ and c-di-GMP on the regulation of biofilm development and flagellar biogenesis using a novel approach based on the screening of a partial *P*. *putida* KT2440 ordered promoter library, containing 94 promoters potentially related to these two processes.

## Materials and Methods

### Bacterial strains and planktonic growth conditions

Bacterial strains used in this work are summarized in Table 1. Planktonic cultures of *Escherichia coli* and *P*. *putida* strains were routinely grown in Luria-Bertani (LB) broth [41] at 37°C and 30°C respectively, with 180 rpm shaking. For high-throughput planktonic bacterial cultures, cells were grown in 250 μl cultures in 2 ml deep 96-well plates (Axygen) at 25°C and 400 rpm shaking in a 2.5 cm stroke radius Innova44 shaker incubator (New Brunswick). For solid media, Bacto-Agar (Difco) was added to a final concentration of 18 g l^-1^. Antibiotics and other additions were used, when required, at the following concentrations: ampicillin (100 mg l^-1^), carbenicillin (0.5 to 1 g l^-1^), kanamycin (25 mg l^-1^), rifampicin (10 mg l^-1^), chloramphenicol (15 mg l^-1^), gentamycin (10 mg l^-1^), tetracycline (5 mg l^-1^), streptomycin (50 mg l^-1^), 5-bromo-4-chloro-3-indoyl-β-D-galactopyranoside (X-gal) (25 mg l^-1^) and sodium salicylate (2 mM). All reagents were purchased from Sigma-Aldrich, except for X-Gal, which was purchased from Fermentas.

**Table 1:** Bacterial strains and plasmids used in this work.

Bacterial strain	Genotype/phenotype	Reference/source
*E*. *coli*		
DH5α	Φ80d*lac*ZΔM15 Δ(*lacZYA-argF*)U169 *recA*1 *endA*1 *hsdR*17 (r_k_^-^ m_k_^+^) *supE*44 *thi*-1 *gyrA relA*1	[42]
NCM631	*hsdS gal* DE3::*lacI lacUV5*::*gen1*(T7 RNA polymerase) Δ*lac* linked to Tn*10*	[43]
*P*. *putida*		
KT2440	mt-2 *hsdR*1 (r^-^ m^+^)	[44]
KT2440*rpoN*	KT2440 *rpoN*::Km. Km^r^	[45]
KT2440 *fliA*::*aphA-3*	KT2440 *fliA*::Km. Km^r^	[46]
KT2442	mt-2 *hsdR*1 (r^-^ m^+^) Rif^r^	[44]
KT2442::miniTn*7*BB-Gm	KT2442 miniTn*7*BB-Gm::*glmS*. Rif^r^ Gm^r^	[47]
KT2442::TpMRB73	KT2442 miniTn*7*BB-Gm[*fleQ*]::*glmS* Rif^r^ Gm^r^	This work
MRB32	KT2442 Δ*bifA*. Rif^r^	This work
MRB34	KT2442 *lapA*::miniTn*5*-Km. Rif^r^ Km^r^	This work
MRB35	KT2442 *fleQ*::miniTn*5*-Km. Rif^r^ Km^r^	This work
MRB35::miniTn*7*BB-Gm	KT2442 *fleQ*::miniTn*5*-Km miniTn*7*BB-Gm::*glmS* Rif^r^ Km^r^ Gm^r^	This work
MRB35::TpMRB73	KT2442 *fleQ*::miniTn*5*-Km miniTn*7*BB-Gm[*fleQ*]::*glmS* Rif^r^ Km^r^ Gm^r^	This work
MRB47	KT2442 *fliG*::miniTn*5*-Km. Rif^r^ Km^r^	This work
MRB50	KT2442 Δ*bifA fleQ*::miniTn*5*-Km Rif^r^ Km^r^	This work
Plasmid	Genotype/phenotype	Reference/source
pENTR^™^/D-TOPO^®^	Gateway^®^ system entry vector for directional cloning. Km^r^	Invitrogen
pIZ227	pACYC184-derived plasmid containing *lacI*^q^ and the T7 lysozyme genes; Cm^r^	[43]
pMPO234	Broad-host range *lacZ* transcriptional fusion vector, based on pBBR1MCS-4. Ap^r^	[48]
pMPO284	pPS854-derived vector containing the miniTn*5-*Km Km^r^ gene flanked by FRT sites, Ap^r^,Km^r^	This work
pMRB1	pMPO234-derived broad-host range *gfp*mut3-*lacZ* transcriptional fusion vector. Ap^r^	[37]
pMRB2	pMRB1-derived vector containing the Gateway^®^ vector conversion *attR1-*Cm^r^*-ccdB-attR2* cassette (forward orientation). Ap^r^, Cm^r^	[37]
pMRB3	pMRB1-derived vector containing the Gateway^®^ vector conversion *attR1*-Cm^r^-*ccdB*-*attR2* cassette (reverse orientation). Ap^r^, Cm^r^	This work
pMRB30	pEX18Tc-derived plasmid with a FRT-Km^r^-FRT cassette between *bifA* upstream and downstream regions. Tc^r^, Km^r^	This work
pMRB73	Delivery plasmid for the miniTn*7*BB-Gm[*fleQ*] transposon, expressing *fleQ* from its promoter region. Ap^r^, Gm^r^	This work
pMRB89	pKT230-derived plasmid expressing *E*. *coli yhjH* from the P*sal* promoter. Sm^r^, Mob^+^	[37]
pMRB90	pMRB30-derived plasmid bearing a Gm^r^ cassette disupting the original Km^r^ cassette. Ap^r^ Gm^r^	This work
pMRB95	pTYB12-based vector for FleQ overproduction. Ap^r^	This work
pMRB99	pSB1K3-derived plasmid expressing *fleQ* from its own promoter. Km^r^	This work
pRK2013	Helper plasmid for triparental mating. ColE1 replicon, Km^r^	[49]
pSB1K3	High copy number cloning vector. Km^r^	[50]
pTNS2	R6K replicon-based Tn*7* transposition helper plasmid. Ap^r^	[51]
pTYB12	Expression vector for N-terminal Intein–chitin-binding domain protein fusions. Ap^r^	New England Biolabs
pUC18Sfi-miniTn*7*BB-Gm	pUC18Sfi-based delivery plasmid for the synthetic transposon miniTn*7*BB-Gm. Ap^r^, Gm^r^	[37]
pYedQ	pRK404A-derived plasmid expressing *E*. *coli yedQ* from the P*lac* promoter. Tc^r^	[52]

### Biofilm growth and quantification

For procedures involving biofilm growth, overnight cultures grown in LB broth were diluted in the same medium to an A_600_ of 0.1 and 150 μl were dispensed into wells of Costar 96 microtiter polystyrene plates (Corning). The plates were incubated at 25°C with moderate shaking (150 rpm) for the desired period of time and processed for planktonic and biofilm growth quantification, essentially as described [53]. Dilution-based planktonic and biofilm growth curves were performed as previously described [47]. For each experiment, at least 3 biological replicates were assayed in octuplicate.

For pellicle and aggregate detection, fresh colonies were inoculated in glass tubes containing 5 ml of 1% bacto-tryptone (Difco) broth [54]. Cultures were incubated overnight at 30°C with shaking, after which the tubes were placed on a rack for 10 minutes and documented by digital photography.

### Plasmid and strain construction

Plasmids used in this work are summarized in Table 1 and S1 Table. Sequences of oligonucleotides used in PCR reactions are available upon request. All DNA manipulations were performed following standard procedures [41]. Restriction and modification enzymes were used according to the manufacturers instructions (Fermentas, Roche and NEB). When required, blunt ends were generated using the Klenow fragment or T4 DNA polymerase. *E*. *coli* DH5α was used as a host in cloning procedures. All cloning steps involving PCR were verified by commercial sequencing (Secugen). Plasmid DNA was transferred to *E*. *coli* and *P*. *putida* strains by transformation [41], triparental mating [55] or electroporation [56]. Site-specific integration of miniTn*7* derivatives was performed essentially as described [51].

Two Gateway^®^ expression vectors, pMRB2 and pMRB3, were constructed to host the ordered promoter library. The *gfp*mut3 allele was PCR-amplified, and the resulting product was cleaved with BamHI and BglII and cloned in the single BamHI site of pMPO234 to yield the *gfp*mut3*-lacZ* fusion vector pMRB1. Then, the Gateway^®^ Vector Conversion System *att*R1-Cm^r^-*ccdB*-*att*R2 cassette was cloned at the SmaI site in pMRB1 in both orientations to generate pMRB2. For construction of a miniTn*7*-based *fleQ* complementation plasmid, a DNA fragment containing the complete *fleQ* and its promoter region was PCR-amplified, the PCR product cleaved with EcoRI and XbaI and ligated into EcoRI- and SpeI- digested pUC18Sfi-miniTn*7*BB-Gm to yield pMRB73.

For generation of a *bifA* deletion in the *fleQ*::miniTn*5*-Km mutant, the gentamycin resistance cassette from miniTn*7*BB-Gm was excised with NcoI and cloned into the sole NcoI site in pMRB30 to disrupt the kanamycin resistance gene, yielding pMRB90. The disruption vectors were transferred to the corresponding hosts (KT2442 or MRB35, respectively) by electroporation. Selection of integration, allelic replacement and FLP-mediated excision of the kanamycin resistance marker was performed essentially as described [57, 58]. The structure of the deleted *bifA* locus was verified by PCR and Southern blot.

For the construction of pMRB95, a plasmid overproducing a chitin binding domain (CBD)-intein-FleQ fusion for protein purification, the *fleQ* open reading frame was PCR amplified and cloned into NdeI- and EcoRI-cleaved pTYB12 (New England Biolabs).

### Construction of an ordered promoter library

For construction of an ordered library of biofilm- and motility-related promoters, intergenic regions of the complete *P*. *putida* KT2440 chromosome sequence [59] harbouring promoters were identified based on experimental evidence in the literature and/or an operon prediction algorithm [Database of prOkaryotic OpeRons (DOOR)] [60, 61] (S1 Table). A subset of promoter-bearing intergenic regions potentially related to motility and/or biofilm development were selected and PCR-amplified using a 5'CACC tag in the forward primer, directionally cloned into pENTR-D/TOPO and subsequently transferred to pMRB2 or pMRB3. According to the orientation of the *att*R1-Cm^r^-*ccdB*-*att*R2 cassette in pMRB2 and pMRB3, forward strand promoters (as displayed in the *P*. *putida* KT2440 genome sequence) were cloned in pMRB2 and reverse strand promoters were cloned in pMRB3 to grant proper orientation relative to the reporter genes. Intergenic regions containing two divergent promoters of interest were cloned in both vectors. The library was constructed and stored in *E*. *coli* DH5α.

### Phase-contrast microscopy

Phase contrast microscopy of surface adhesion was performed on a Leica DMI4000B inverted microscope using 20x objective and 1.6x ocular magnification. Cells from mid-exponential (A_600_ = 0.2–0.3) LB cultures of the selected *P*. *putida* strains were serially diluted (10^2^−10^4^) in LB and samples were transferred to wells of Costar 96 microtiter polystyrene plates (Corning). Attachment was allowed to proceed for 30 minutes, planktonic cells were removed by washing twice with 150 μl LB, and then 50 μl LB was added to the wells. For short-term assessment of surface attachment, plates were incubated at room temperature for 3 hours, and then cells deposited on the plane of the well surface were video recorded for 1 minute. For long-term assessment of surface attachment, a field containing a single cell was selected and recorded in a time-lapse series at 2-minute intervals during 16 hours.

### Swimming and swarming assays

Swimming assays were adapted from [62]. Tryptone motility plates containing 0.3% Bacto-agar (Difco) were toothpick-inoculated with fresh colonies and incubated for 12 h at 30°C. Digital photographs were taken, and the swimming zone diameter was measured and normalized that of the wild-type. At least 3 biological replicates were assayed for each strain.

Swarming assays were performed essentially as described by [63], with some modifications. Two and a half microliters of overnight LB cultures were spotted onto the center of plates containing 0.5% PG-agar [proteose peptone No. 3 (Difco 212693), 0.5%, and glucose, 0.2%, with Difco Bacto-Agar] and plates were incubated at 25°C for 40 hours. At least two biological replicates per experiment were assayed in three separate experiments.

### Congo Red (CR) binding assay

To assess cellulose-like polysaccharide production the CR binding assay was adapted from [54]. Bacterial cells from 2 ml overnight cultures in LB were collected by centrifugation and resuspended in 1 ml 1% bacto-tryptone (Difco) broth containing 40 μg/ml CR. The mixtures were incubated at 30°C with shaking at 180 rpm for 180 min. The bacterial cells and bound CR were removed by centrifugation (1 min, 13000 rpm), and the amount of CR remaining in the supernatants was determined by measuring the absorbance of the supernatant at 490 nm, using the same medium supplemented with 40 μg/ml CR as a control.

### Promoter library screening

For screening the ordered promoter library, the fusion plasmids were transferred to the appropriate *P*. *putida* strains by mating. Exconjugant colonies were inoculated in deep-well microtiter plate wells containing LB and grown overnight as described above. Cultures were diluted 100-fold and re-grown for 24 h in the same conditions, except that 2 mM salicylate was added to the strains bearing pMRB89. Gene expression was assessed by GFP fluorescence measurements. To this end, 200 μl culture samples were transferred to Costar 96-well microtiter polystyrene plates to determine the A_600_, from which 150 μl were subsequently transferred to Microfluor 1 Black 96-well plates (Thermo LabSystems) to register fluorescence using a 485 nm band pass excitation filter and a 520 nm emission filter on a POLARstar Omega Fluorometer (BMG Labtech). Specific fluorescence was calculated by dividing the fluorescence reading by the A_600_ reading.

### β-galactosidase assays

For β-galactosidase assays of *lacZ* fusions, overnight cultures bearing the corresponding fusion plasmids were diluted in 5 ml LB to an A_600_ of 0.01 and incubated for 24 hours at 30°C with shaking. Growth was then stopped, and β-galactosidase activity was determined from sodium dodecyl sulfate- and chloroform-permeabilized cells as described [64].

#### RNA preparation and real time qRT-PCR

Total RNA was prepared from stationary phase cultures as previously described [65]. Reverse transcription (RT) of total RNA (5 μg) was carried out using the high-capacity cDNA Archive kit (Applied Biosystems), with random hexamers as primers. Negative and positive controls were performed with total RNA or genomic DNA as templates, respectively. qPCR was performed using specific primers for the *lapA* and *bcsD* coding sequences and SYBR Green technology in an ABI Prism 7000 Sequence Detection System (Applied Biosystems) essentially as described [66].

### FleQ purification and gel mobility shift assays

FleQ was overproduced from the T7 promoter as a N-terminal CBD-intein fusion. T7 RNA polymerase-expressing strain NCM631 bearing pIZ227 was used as a host. Overproduction and purification using the IMPACT kit, featuring chitin affinity chromatography followed by intein self-cleavage induced by reducing conditions, were performed essentially as described for *P*. *aeruginosa* FleQ [13]. The purified protein was dialized against FleQ storage buffer (20 mM Tris-HCl, pH 8.0; 250 mM KCl; 0.1 mM EDTA; 2 mM DTT; 50% glycerol), and stored in aliquots at -80°C. Protein concentration was determined by means of the Bio-Rad Protein assay (Bio-Rad), and purity was assessed by SDS-PAGE.

Gel mobility shift assays were performed essentially as described [13]. The P*cdrA*, P*pp1155*, P*lapA* and P*bcsD* promoter regions were PCR amplified from pCdrA^C^, and clones PP1155, PP0168 and PP2629 from the promoter library, repectively. The resulting products were subsequently cleaved with appropriate restriction enzymes to yield fragments <500 bp. The cleaved probes were challenged with different FleQ concentrations in a binding buffer containing 10 mM Tris, pH 7.8; 8 mM magnesium acetate; 50 mM KCl; 5% glycerol, and 250 ng ml^-1^ BSA for 30 minutes at room temperature, resolved in native 5% PAGE at 4°C, stained with ethidium bromide and documented by digital photography.

## Results

### FleQ is required for flagellar motility, surface attachment and biofilm formation

**Fig 1:**
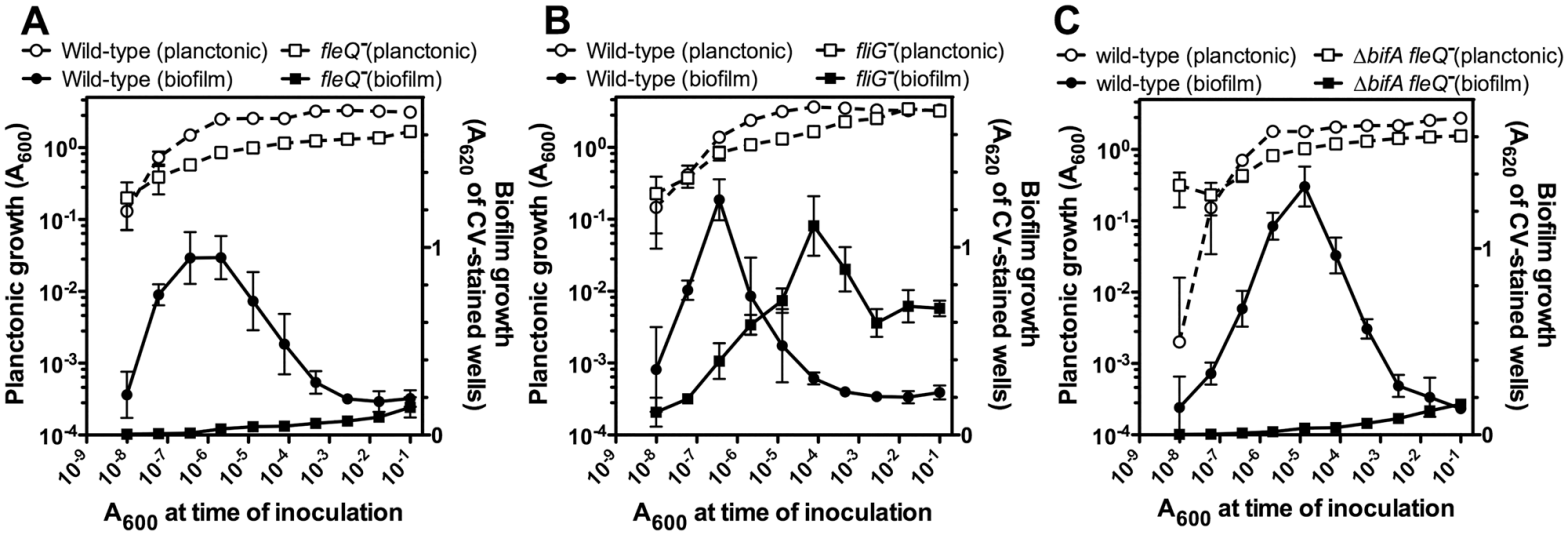
Dilution series-based growth curves of KT2442 derivatives. Planktonic (left axes, open symbols) or biofilm growth (right axes, closed symbols) is plotted against the initial A_600_ of each dilution. Circles represent the wild-type KT2442 strain and squares represent the *fleQ* mutant MRB35 (**A**), the *fliG* mutant MRB47 (**B**), or the Δ*bifA fleQ* mutant MRB50 (**C**). Plots display one representative experiment of at least three biological replicates. Error bars represent the standard deviation of the six technical replicates.

In order to characterize the contribution of FleQ to the biofilm developmental cycle of *P*. *putida*, we examined the biofilm development kinetics of strains KT2442 (wild-type) and MRB35 (*fleQ*) in LB by means of a serial dilution-based growth curve method we described recently, in which a dilution series is used to recapitulate the time course of planktonic and biofilm growth in microtiter plate wells [47] (Fig 1A). The wild-type strain displayed a steady increase in biofilm growth to reach a maximum coincident with the onset of stationary phase, and then biofilm biomass decreased, consistent with nutrient starvation-induced biofilm dispersal in this organism [47]. In contrast, surface-attached biomass of the *fleQ* mutant MRB35 was negligible during the whole growth curve. Complementation with a site-specifically inserted miniTn*7* transposon derivative expressing FleQ from its natural promoter restored a normal biofilm formation and dispersal pattern in MRB35 (S1 Fig). These results strongly suggest that FleQ integrity is critical for biofilm formation.

The *fleQ* mutant is non-motile (S2 Fig), as expected from its assumed role as a regulator of flagellar biogenesis. On the other hand, the lack of flagella has been shown to correlate with surface adhesion defects in *P*. *putida* [67–69]. Thus, to clarify whether the biofilm formation defect of the *fleQ* mutant is related to the lack of flagellar motility, the evolution of planktonic and biofilm growth of the non-motile mutant MRB47, bearing a miniTn*5*-Km insertion in *fliG*, which encodes a flagellar motor protein, was analyzed by means of dilution series-based growth curves as above (Fig 1B). Planktonic and biofilm growth of MRB47 was slow compared to that of the wild-type strain. However, this phenotype was relatively mild compared to that of the *fleQ* mutant (Fig 1A), as substantial levels of biofilm biomass were achieved. Analysis of other non-motile mutants bearing insertions in the flagellar structural genes *flgG*, *fliF* and *fliN* and *fliP* yielded similar results [40]. These results indicate that the loss of flagellar motility does not suffice to explain the severe biofilm formation defect of the *fleQ* mutant.

**Fig 2:**
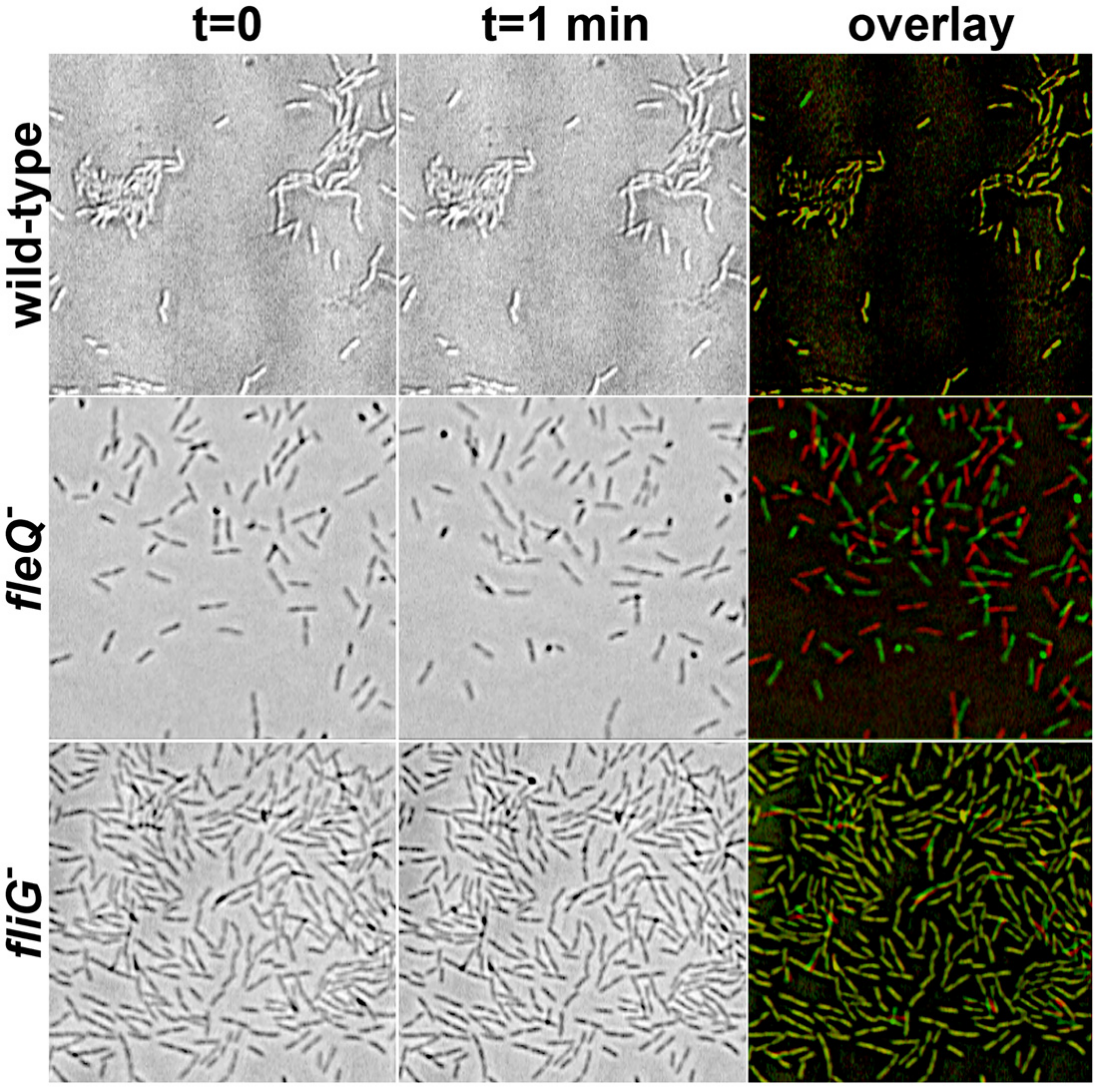
Adhesion assays of KT2442 derivatives. Phase-contrast micrographs of the wild-type strain KT2442 (top) and its *fleQ and fliG* mutant derivatives MRB35 (center) and MRB47 (bottom). Two frames of the same field were taken in a 1-minute interval (left and center). The right panel shows an overlay of the two images of each strain digitally colored red (t = 0) or green (t = 1 min).

To determine whether the lack of FleQ has an impact on bacterial adhesion to surfaces, we used phase-contrast microscopy to analyze the adhesion phase of biofilm formation. To this end, cells of the wild-type, *fleQ* and *fliG* strains were allowed to attach to the bottom of polystyrene microtiter plate wells, and were then recorded in 1-minute videos (Fig 2). Observation of the images obtained revealed that the majority of the wild-type and *fliG* cells were immobilized on the polystyrene surface, strongly suggesting that both strains are capable of irreversible attachment. In contrast, the *fleQ* mutant cells displayed characteristic Brownian motion and were not immobilized in these conditions. Furthermore, monitoring the evolution of a single *fleQ* cell offspring for 16 hours in a 2-minute time-lapse movie failed to reveal any significant attachment (data not shown), strongly suggesting that FleQ is absolutely required for strong, irreversible interaction with the surface. Taken together, these results indicate that the lack of flagellar motility does not impair surface attachment, and therefore the strong adhesion defect of the *fleQ* mutant must be due to an additional role of FleQ in the regulation of cell-surface interactions.

### FleQ is required for multiple phenotypes associated with high c-di-GMP levels

Increased c-di-GMP levels have been shown to stimulate surface adhesion and biofilm formation in *P*. *putida* [33, 63]. We have recently described that mutational inactivation of *bifA*, encoding a c-di-GMP phosphodiesterase, results in elevated intracellular c-di-GMP concentration [37]. To test whether high c-di-GMP levels may boost biofilm formation in the absence of *fleQ*, the Δ*bifA fleQ* mutant MRB50 was constructed and its biofilm development kinetics was assessed as above. The behavior of MRB50 was indistinguishable from that of the *fleQ* mutant MRB35 (Fig 1C), strongly suggesting that FleQ is required for c-di-GMP-dependent stimulation of biofilm growth.

**Fig 3:**
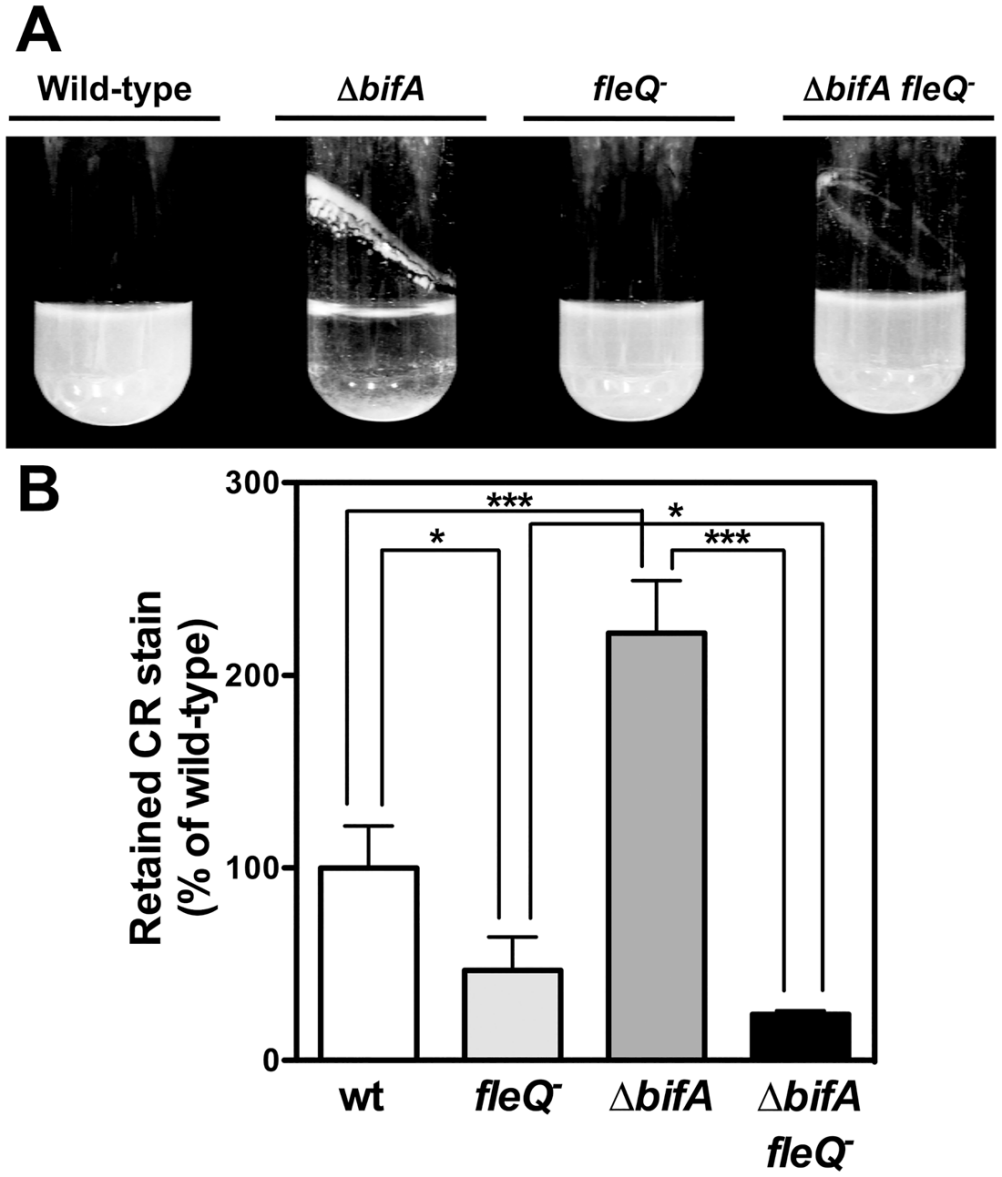
Pellicle, aggregation and CR adsorption phenotypes of Δ*bifA* and *fleQ* mutants. **A.** Photographs of planktonic cultures of the wild-type (KT2442), Δ*bifA* (MRB32), *fleQ* (MRB35) and Δ*bifA fleQ* (MRB50) strains showing pellicle formation and culture clarification due to aggregation. **B.** CR adsorption of the wild-type (KT2442), Δ*bifA* (MRB32), *fleQ* (MRB35) and Δ*bifA fleQ* (MRB50) strains. Data are normalized to the wild-type, set to 100%. Bars represent the averages and standard deviations of at least three independent assays.

Increased c-di-GMP levels have also been shown to promote cell aggregation in liquid medium, biofilm formation in the medium-air interphase (pellicle) and increased adsorption of Congo Red (CR) to the cell surface [37, 63]. These phenotypes were also tested in the wild-type strain KT2442 and its Δ*bifA*, *fleQ* and Δ*bifA fleQ* derivatives MRB32, MRB35 and MRB50 (Fig 3). The wild-type and *fleQ* mutant strains showed little pellicle biomass and no detectable aggregation (Fig 3A). In contrast, most of the Δ*bifA* mutant biomass was in the form of glass-attached pellicle and cell aggregates, consistent with the high c-di-GMP levels present in this strain. Interestingly the double Δ*bifA fleQ* mutant MRB50 did not display an aggregative behavior, and showed decreased levels of pellicle formation. On the other hand, the Δ*bifA* mutant MRB32 bound twice as much CR as the wild-type (Fig 3B). In contrast, CR retention by the *fleQ* mutant MRB35 was 2-fold reduced relative to the wild-type. CR retention by the double Δ*bifA fleQ* mutant MRB50 was also decreased (4-fold). The fact that FleQ is required for an array of phenotypes generally associated to sessile growth and induced by high intracellular c-di-GMP concentrations strongly suggests the involvement of FleQ in c-di-GMP regulation of adhesion and biofilm growth.

### FleQ and c-di-GMP coordinately regulate motility- and biofilm-related promoters

**Fig 4:**
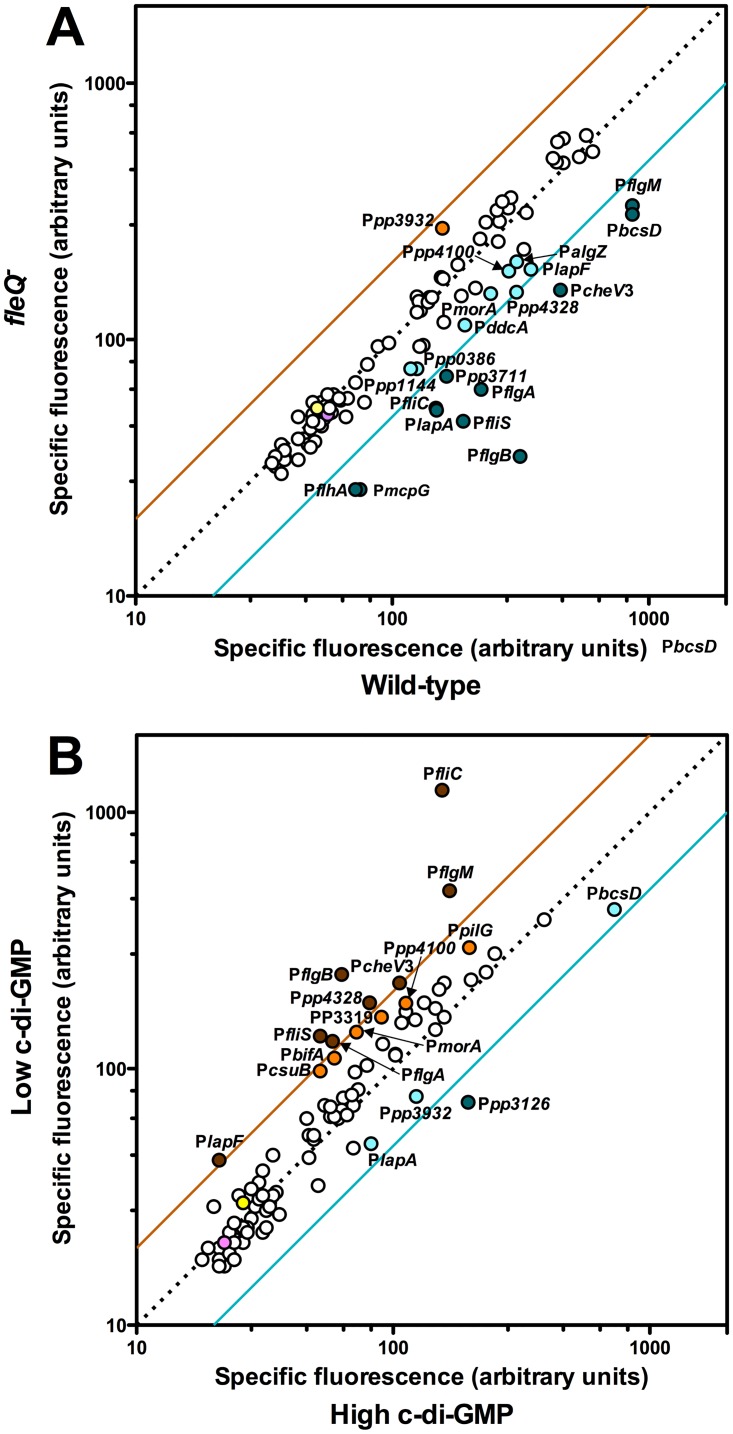
Screenings of the ordered promoter library for FleQ and c-di-GMP regulation. Diagonal plots showing expression of each promoter fusion in the *fleQ* (MRB35) vs. wild-type (KT2442) backgrounds (**A**), or in the presence of low vs. high levels of c-di-GMP (**B**). Red and blue lines represent 2-fold differential expression. Data points showing over 2-fold differential expression are shown in dark red or dark blue. Data points showing over differential expression greater than 1.5-fold but lower than 2-fold are shown in orange or cyan. Data from the experimental controls (KT2442 and KT2442 bearing the empty vector pMRB1) are shown in yellow and pink,respectively. Promoters selected for confirmation are indicated in each plot. The original data for these plots are shown in S2 Table.

To determine the extent of FleQ-mediated regulation of *P*. *putida* lifestyle changes, we screened a library of 94 *P*. *putida* KT2440 promoters fused to the reporters *gfp*mut3 and *lacZ*. This library includes promoters directing the synthesis of adhesion factors, extracellular matrix components, appendages, and known or assumed regulatory elements of biofilm development and motility, such as a variety of transcription factors and potential c-di-GMP DGC or PDE proteins (See Materials and Methods and S1 Table for details). To this end, the library fusion plasmids, along with the empty vector pMRB1, were transferred to the wild-type *P*. *putida* KT2442 and its *fleQ* derivative MRB35, and expression was determined from fluorescence measurements of stationary phase planktonic LB cultures grown in deep-well microtiter plate wells (Fig 4A).

**Fig 5:**
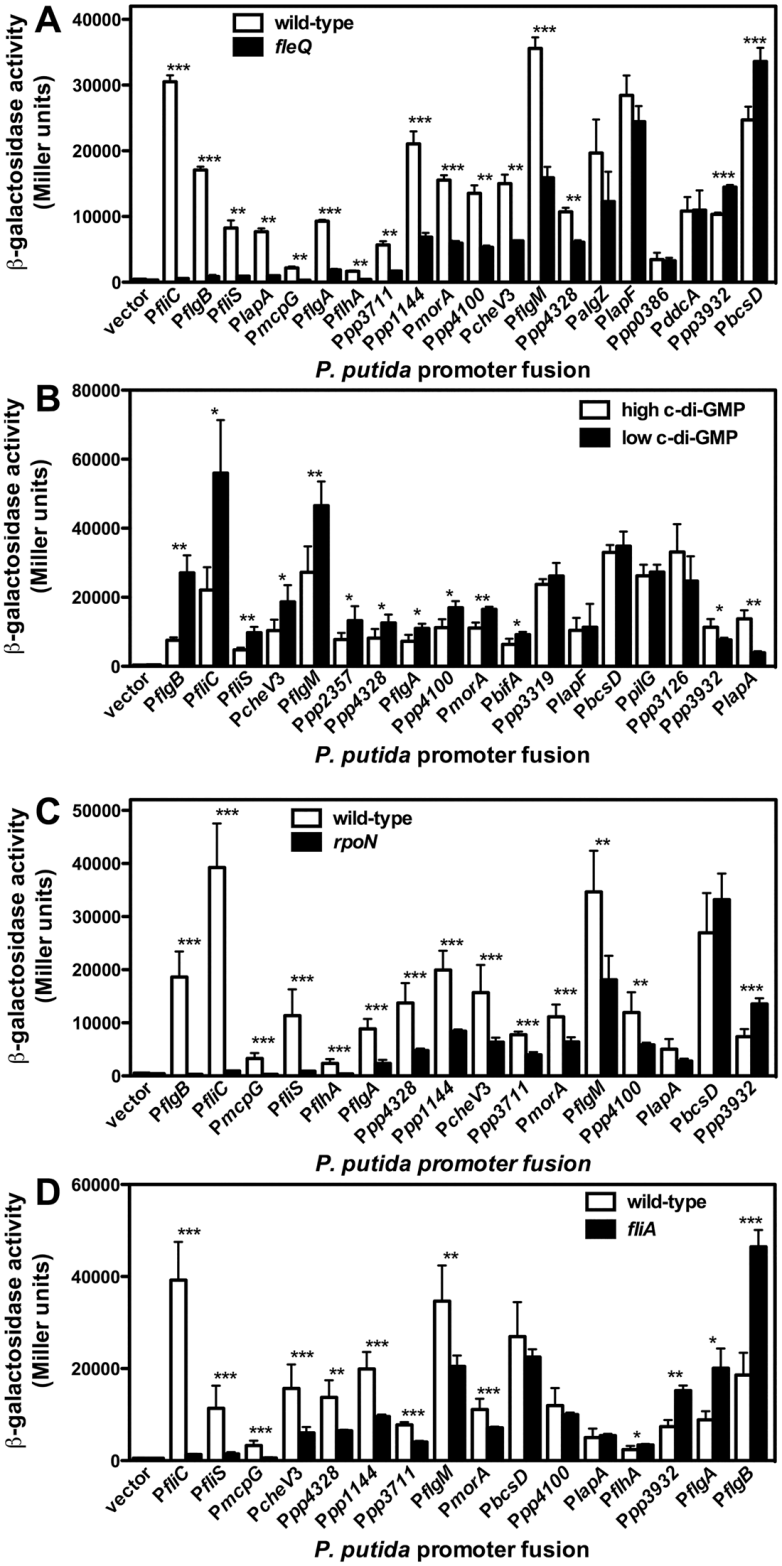
Expression of the selected promoters in different *P*. *putida* backgrounds. β-galactosidase assays of the selected promoter fusions in wild-type (KT2442) and *fleQ* (MRB35) backgrounds (**A**), a *lapA*^*-*^ mutant (MRB34) bearing pYedQ (high c-di-GMP) or pMRB89 (low c-di-GMP) (**B**), wild-type (KT2440) and *rpoN*^*-*^ (KT2440*rpoN*) backgrounds (**C**), and wild-type (KT2440) and *fliA* (KT2440*fliA*::*aphA-3*) backgrounds (**D**) Bars represent the averages and standard deviations of at least three independent assays. Stars designate p-values for the Student's *t*-test for unpaired samples not assuming equal variance. *: p<0.05; **: p<0.01; ***:p<0.005.

Twenty promoters of the library, displaying at least 1.5-fold differential expression in the *fleQ* mutant relative to the wild-type strain, were initially chosen for further analysis. All but one promoter were upregulated in the wild-type relative to the *fleQ* mutant, suggesting that FleQ acts preferentially as an activator in *P*. *putida*. For confirmation, individual cultures of the wild-type and *fleQ* strains bearing the FleQ-regulated fusions obtained in the screening were re-tested by means of β-galactosidase assays. Sixteen promoters were confirmed to show significant FleQ-dependent regulation (p<0.05) (Fig 5A). The set included seven promoters of the flagella/chemotaxis gene cluster, located upstream from *fliC*, *flgB*, *fliS*, *flgA*, *flhA*, *cheV*3 and *flgM* and predicted to transcribe the *fliC-fleL*, *flgBCDE-pp4387*, *fliST*, *flgA*, *flhAF-fleN-fliA*, *cheV*3*R* and *flgMN* operons, and additional promoters upstream from PP4328, predicted to transcribe the *pp4328-4329* operon, encoding a FliK homolog and a FlhB domain protein, and *mcpG*, encoding a gamma-amino butyric acid-responsive methyl-accepting chemotaxis protein (MCP)[70]. All these promoters were positively regulated by FleQ to various extents, ranging from 1.8-fold (P*pp4328*) to 54-fold (P*fliC*). These results support the notion that FleQ is the master regulator of the synthesis of the flagellar motility and chemotaxis apparatus in *P*. *putida*. Two additional FleQ-regulated promoters, P*lapA*, directing the synthesis of the high molecular weight adhesin LapA, and P*bcsD*, predicted to direct transcription of the *bcsDEFGRQABZC* operon, encompassing the cellulose synthesis and export *bcs* genes, were related to the biofilm lifestyle [28, 38]. While P*lapA* was positively regulated (8-fold), P*bcsD* displayed modest negative regulation (~1.5-fold) by FleQ. It should be noted that P*bcsD* was initially picked in the screening as a FleQ-activated promoter. However, further verification indicated that the expression levels obtained in the screening were atypical, and confirmed negative regulation of this promoter. Promoters upstream from PP1144, PP3711 and *morA*, encoding GGDEF/EAL proteins were positively regulated (~3-fold) by FleQ, while P*pp3932*, directing the synthesis of a putative GGDEF domain protein was negatively regulated, albeit modestly (1.4-fold). Finally, P*pp4100*, predicted to direct transcription of the *pp4100-gacA-uvrC-pgsA* operon, encoding a Cro/cI family regulator, response regulator GacA, ABC excinuclease C subunit and CDP-diacylglycerol-glycerol-3-phosphate 3-phosphatidyltransferase, was 3-fold upregulated by FleQ, indicating that GacA, a regulatory element known to be relevant to biofilm formation in *P*. *aeruginosa* [71], is also a part of the FleQ regulon. These results are consistent with the phenotypic analysis above showing that FleQ is required for both flagellar motility and biofilm development, and suggests that it may do so by means of a complex network involving additional regulators and modulation of c-di-GMP synthesis and hydrolysis.

Similarly, the promoter library was screened for differential expression in response to changes in c-di-GMP levels. To this end, we overproduced the *E*. *coli* DGC YedQ or the PDE YjhH from plasmids pYedQ or pMRB89, to increase or decrease, respectively, the intracellular c-di-GMP concentration. In pMRB89 *yhjH* is under control of the P*sal* promoter, and expression was induced by adding 2 mM salicylate to the growth medium. In pYedQ, *yedQ* is transcribed from the P*lac* promoter. However, IPTG-induced YedQ overexpression inhibited growth and even in the absence of IPTG, YedQ production provoked strong aggregation that hampered fluorescence measurements (data not shown). Thus, the non-aggregating *lapA*^*-*^ derivative of KT2442 MRB34 was used in this experiment, and IPTG was not added to the growth medium. Accordingly, the library fusion plasmids, along with the empty vector pMRB1, were transferred to MRB34 bearing pYedQ or pMRB89, and expression was determined from fluorescence measurements as above (Fig 4B).

**Fig 6:**
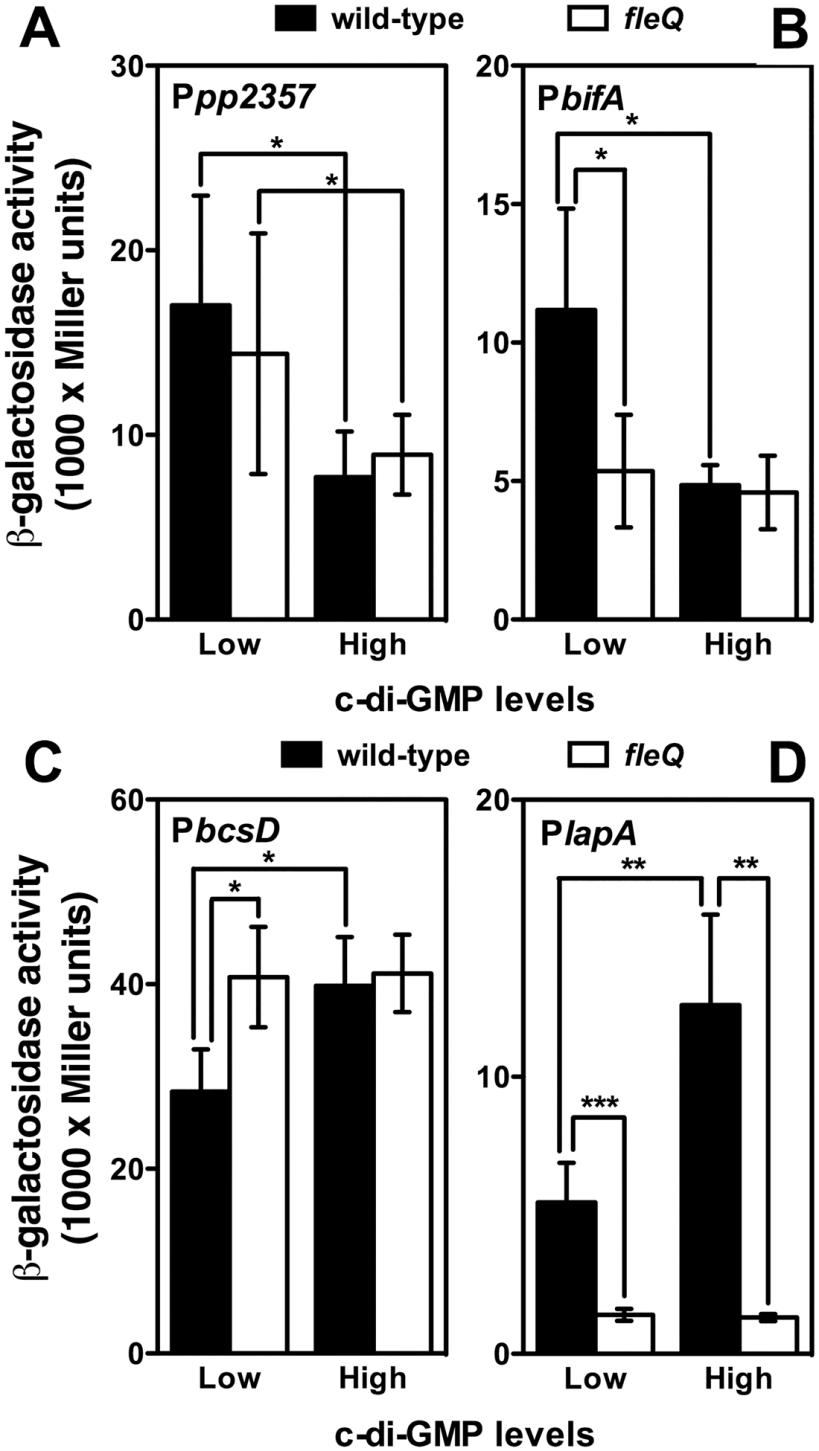
Assessment of combined FleQ and c-di-GMP regulation at selected promoters. β-galactosidase assays of the P*pp2357* (**A**), P*bifA* (**B**), P*bcsD* (**C**) and P*lapA* (**D**) promoter fusions in the wild-type KT2442 and *fleQ* (MRB35) strain bearing the YhjH-producing plasmid pMRB89 (low c-di-GMP) or the YedQ-producing plasmid pYedQ (high c-di-GMP). Bars represent the averages and standard deviations of three independent assays. Stars designate p-values for the Student's *t*-test for unpaired samples not assuming equal variance. *: p<0.05, **: p<0.01; ***: p<0.001.

Eighteen promoters displaying >1.5-fold differential expression in response to changes in the c-di-GMP levels were chosen for further analysis, fourteen of which were repressed by c-di-GMP and four were induced. Thirteen of these promoters were confirmed to be significantly regulated by c-di-GMP by means of β-galactosidase assays, as above (Fig 5B). Interestingly, a great deal of overlap was observed between the set of FleQ-regulated and the set of c-di-GMP-regulated promoters (Fig 6). Nine FleQ-activated promoters were downregulated in the presence of c-di-GMP. This set includes flagella and motility-related promoters (P*fliC*, P*flgB*, P*fliS*, P*flgA*, P*cheV*3, P*flgM* and P*pp4328*), as well as P*morA* and P*pp4100*. The P*lapA* promoter was upregulated by both FleQ and c-di-GMP, while P*pp3932* was downregulated by FleQ and upregulated by c-di-GMP. Two additional promoters, P*pp2357*, predicted to direct transcription of the type I pili biogenesis operon *pp2357-pp2358-2359-2360-csuCDE*, and P*bifA*, directing the synthesis of the PDE BifA, involved in starvation-induced dispersal [37], were negatively regulated by c-di-GMP in a FleQ-independent fashion. Of these two, at least P*bifA* is expected to be FleQ-regulated, as it was shown to be dependent on the flagellar σ factor FliA [46]. Since FleQ responds to changes in the c-di-GMP levels, we also tested FleQ regulation by comparing the expression of the corresponding fusions in the wild-type and *fleQ* strains bearing plasmids pMRB89 or pYedQ (Fig 6). Expression of the P*pp2357* promoter was 2-fold repressed by c-di-GMP in both the wild-type and *fleQ* backgrounds, thus confirming that this promoter is FleQ-independent (Fig 6A). In contrast, 2-fold repression of the P*bifA* promoter by c-di-GMP was only observed in the wild-type background, while the *fleQ* mutant displayed basal expression levels regardless of the c-di-GMP concentration (Fig 6B). These results indicate that P*bifA* is positively regulated by FleQ, but such regulation is only observed at low c-di-GMP concentrations.

### σ factor dependency of FleQ-regulated promoters

FleQ is an enhancer-binding protein known to activate flagellar promoters dependent on the alternative σ factor σ^N^. On the other hand, FleQ has also been shown to indirectly regulate transcription from promoters dependent on the flagella-specific alternative σ factor FliA [10]. To address the σ factor dependence of our FleQ-regulated promoter set, expression from the *gfp-lacZ* fusions was assessed from the wild-type strain KT2440 (the parent strain of KT2442), its *rpoN* mutant derivative KT2440*rpoN* and its *fliA* mutant derivative by means of β-galactosidase assays of stationary phase LB cultures (Fig 5C and 5D).

All nine flagellar motility and chemotaxis-related promoters were upregulated in the wild-type strain relative to the *rpoN* mutant. Promoters P*flgB*, P*fliC*, P*mcpG*, P*fliS* and P*flhA* displayed strict dependency on the alternative σ factor, as their basal expression levels in the *rpoN* mutant were comparable to those in the vector construct and expression was elevated 5- to 58-fold (for P*flhA* and P*flgB*, respectively) in the presence of σ^N^. A lesser extent of regulation (2- to 3-fold) and higher σ^N^-independent basal levels were observed for P*flgA*, P*pp4328*, P*cheV*3 and P*flgM*, indicating partial σ^N^-dependence of these promoters. The latter pattern was also observed with the P*pp1144*, P*pp3711*, P*morA* promoters, driving the synthesis of three GGDEF/EAL domain proteins, and P*pp4100*, directing the synthesis of the transcription factor GacA and other proteins, while P*pp3932* was inversely regulated: its expression was increased 2-fold in the absence of σ^N^. In contrast, P*lapA* and P*bcsD*, responsible for the production of biofilm matrix components were not affected by the *rpoN* mutation.

Six of the flagellar motility and chemotaxis-related promoters were upregulated in the wild-type strain relative to the *fliA* mutant. Promoters P*fliC*, P*fliS* and P*mcpG* displayed strict dependency of the alternative σ factor, as their basal expression levels in the *fliA* mutant were comparable to those in the vector construct and expression was elevated 4- to 30-fold (for P*mcpG* and P*fliC*, respectively) in the presence of FliA. A lesser extent of regulation (2- to 3-fold) and higher FliA-independent basal levels were observed for P*cheV*3, P*pp4328*, and P*flgM*, indicating partial FliA-dependence of these promoters. The latter pattern was also observed with the P*pp1144*, P*pp3711* and P*morA* promoters. High basal expression in the *fliA* mutant suggests that these promoter regions may also be recognized by RNA polymerase loaded with a σ factor other than FliA, but the biological significance of this is unclear. Expression of three flagella-related promoters (P*flhA*, P*flgA* and P*flgB*), the two biofilm matrix-related promoters P*lapA* and P*bcsD*, P*pp4100* and P*pp3932* was not stimulated by the presence of FliA, and three of them (P*pp3932*, P*flgA* and P*flgB*) were moderately (2-fold) downregulated in the wild-type relative to the *fliA* mutant. Taken together, our results strongly suggest that FleQ is a high-ranked regulator in a cascade that involves the transcriptional regulation of σ^N^-dependent, FliA-dependent and σ^N^- and FliA-independent promoters in *P*. *putida*. In addition, σ^N^- and FliA-dependency appear to be associated with flagellar motility and chemotaxis-associated functions, while synthesis of biofilm matrix components is regulated by FleQ in a σ^N^- and FliA-independent fashion.

#### FleQ directly regulates a subset of flagellar promoters

**Fig 7:**
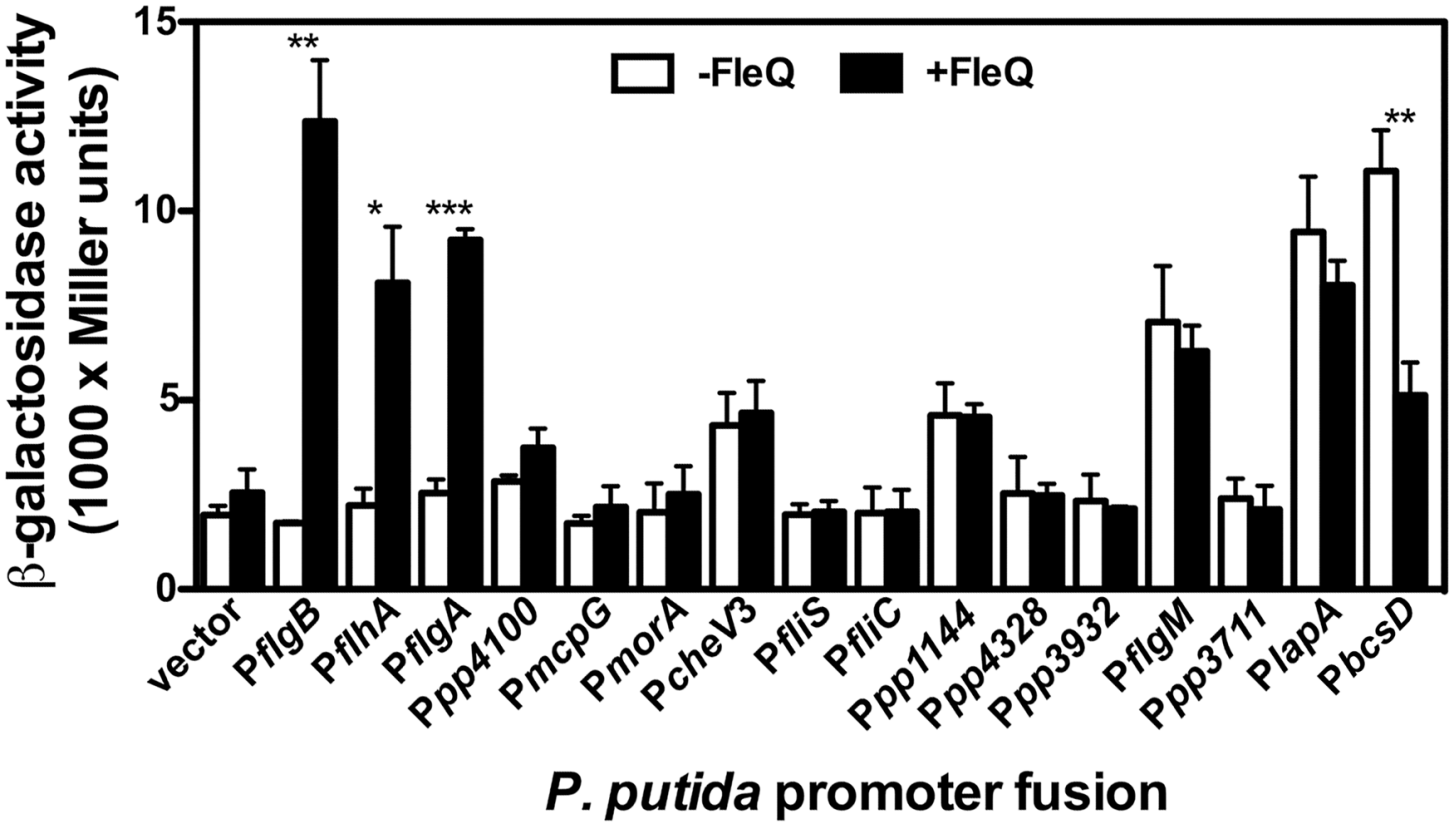
Assessment of direct regulation in FleQ-regulated *P*. *putida* promoters. β-galactosidase assays of the selected promoter fusions in ET8000 bearing the FleQ-producing plasmid pMRB99 (+FleQ) or the empty vector pSB1K3 (-FleQ). Bars represent the averages and standard deviations of three independent assays. Stars designate p-values for the Student's *t*-test for unpaired samples not assuming equal variance. *: p<0.05; **: p<0.01; ***:p<0.005.

To determine whether FleQ-dependent regulation of the *P*. *putida* target genes identified is direct or indirect, plasmids bearing *gfp*mut3-*lacZ* fusions to the complete set of FleQ-regulated promoters, along with the empty vector pMRB1 were transformed into *E*. *coli* ET8000, a heterologous background not encoding a FleQ ortholog, and ET8000 bearing the FleQ-producing plasmid pMRB99, and expression was assessed by means of β-galactosidase assays of LB-grown stationary phase cultures (Fig 7). Expression from the P*flgB*, P*flhA* and P*flgA* fusions was increased 4- to 7-fold in the presence of FleQ, indicating that FleQ directly activates transcription from these three promoters. In contrast, no significant change in expression was observed with the FliA-regulated P*fliC*, P*fliS*, P*flgM*, P*cheV*3, P*pp4328*, P*cheV*3, P*pp4100*, P*mcpG*, P*morA*, P*pp1144*, P*pp3711* and P*pp3932* promoters, consistent with the notion that the effect of FleQ on these promoters is indirect, and mediated by FleQ control of the flagellar cascade.

#### FleQ and c-di-GMP regulation of the P*lapA* and P*bcsD* promoters

For a deeper understanding of the role of FleQ and c-di-GMP in the regulation of biofilm development, we studied further the regulation of the P*lapA* and P*bcsD* promoters. To this end, we assessed the observed FleQ-dependent regulation by qRT-PCR. Assays performed with cDNA obtained from the wild-type and *fleQ* strain showed that the *lapA* mRNA levels were 2-fold (p<0.01) greater in the wild-type strain relative to the *fleQ* mutant, while *bcsD* mRNA levels were 32-fold (p<0.05) elevated in the *fleQ* mutant relative to the wild-type. These results confirm that FleQ is a positive regulator of P*lapA* and a negative regulator of P*bcsD*. In addition, we evaluated the combined effect of *fleQ* and c-di-GMP regulation by assaying expression from the P*lapA* and P*bcsD* fusions in the wild-type and *fnr* strains bearing the YedQ- and YhjH-producing plasmids pYedQ and pMRB89, as shown above for P*pp2357* and P*bifA* (Fig 6). Under a low c-di-GMP regime, P*bcsD* expression was 1.4-fold increased in the absence of FleQ (Fig 6C). However, when c-di-GMP levels were elevated, P*bcsD* expression was high and unresponsive to FleQ. These results suggest that P*bcsD* is repressed by FleQ at low c-di-GMP concentrations, and high c-di-GMP levels antagonize FleQ-dependent repression. In contrast, P*lapA* expression was low in the *fleQ* mutant, and was elevated 4- and 10- fold in the wild-type strain under the low and high c-di-GMP regimes, respectively (Fig 6D), suggestiing that FleQ is an activator of P*lapA* transcription, and high c-di-GMP levels further stimulate FleQ-dependent activation.

**Fig 8:**
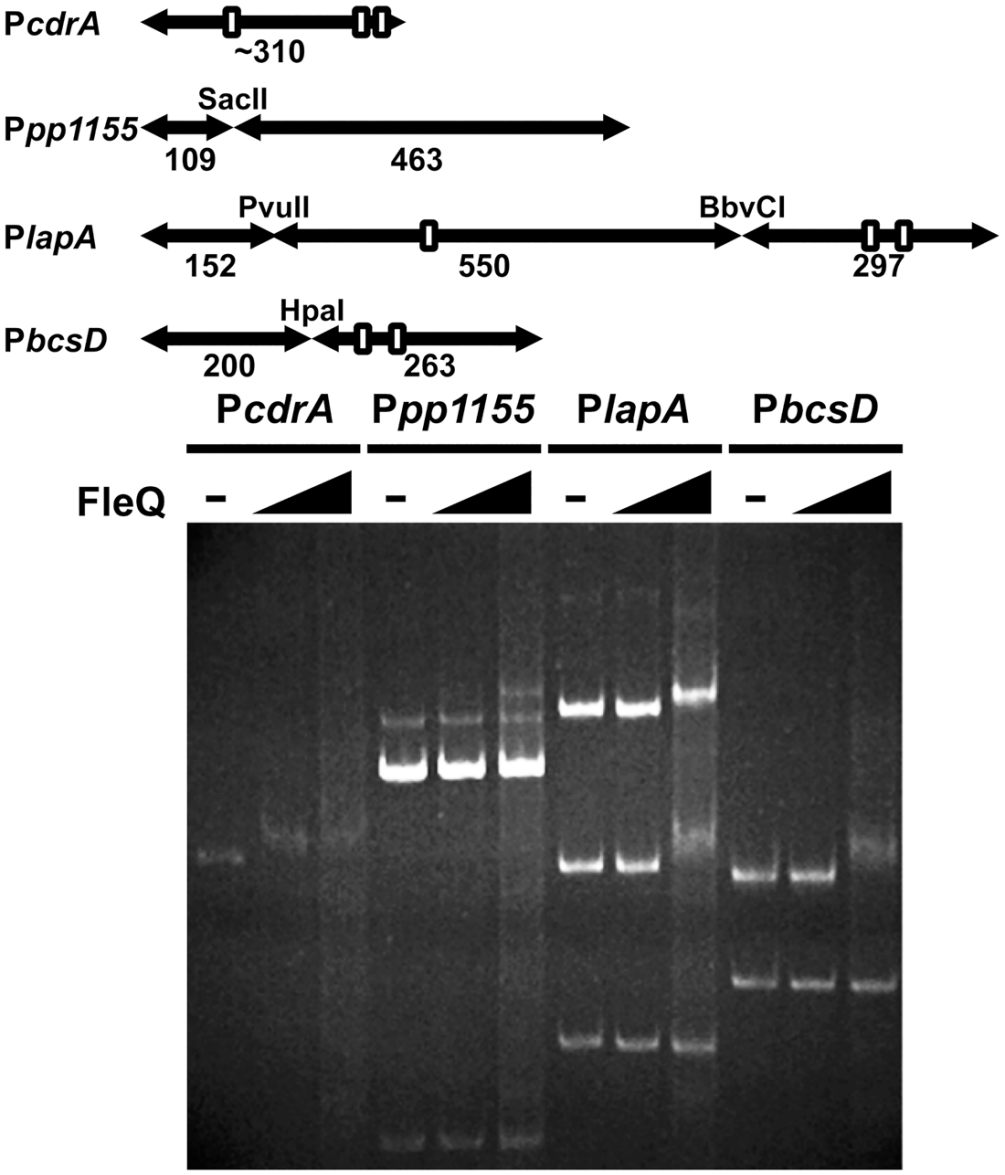
Gel mobility shift assays of FleQ on the P*lapA* and P*bcsD* promoters. **Top:** Cartoon of the probes used for each of the promoter regions, indicating the restriction enzymes used for cleavage, the sizes (in bp) of the resulting fragments, and the location of the predicted FleQ binding motifs (open boxes). Drawn to scale. **Bottom:** Ethidium bromide-stained PAGE showing the results of a typical gel mobility shift assay. Each promoter region was challenged with 0, 0.45 and 4.5 μM FleQ.

We also used the heterologous *E*. *coli* ET8000 background, as shown above, to assess whether FleQ regulation of the two biofilm-related promoters is direct or indirect (Fig 7). When assayed in these conditions, the P*bcsD* promoter was 2-fold downregulated in the presence of FleQ, suggesting that FleQ is a direct regulator of P*bcsD* transcription. However, no regulation was observed with the P*lapA* promoter. While this result may suggest that the effect of FleQ on P*lapA* is indirect, alternative explanations, such as the requirement for additional *P*. *putida* factors other than FleQ for *in vivo* P*lapA* activation, are compatible with direct regulation of P*lapA*. Recently, a consensus sequence for the *P*. *aeruginosa* FleQ binding motif was derived from the analysis of thirteen verified FleQ binding sites [18]. Bioinformatics search for this motif using the FIMO software [72] revealed three highly significant (*P*<10^−4^) matches at the P*lapA* promoter region and two at P*bcsD* (S3 Table), suggesting that FleQ may bind these two regions directly. Finally, to clarify this issue, we resolved to determine the ability of FleQ to interact with the P*lapA* and P*bcsD* promoter regions by means of gel mobility shift analysis. To this end, *P*. *putida* KT2440 FleQ was overproduced as a N-terminal CBD-intein-FleQ fusion, purified by chitin affinity chromatography, and the tag was subsequently self-cleaved to release the native FleQ protein (S3 Fig). Activity of this FleQ preparation was initially tested in non-radioactive gel mobility shift assays with the PCR-amplified *P*. *aeruginosa* P*cdrA*, bearing three experimentally verified FleQ binding sites [18], as a probe (Fig 8). This P*cdrA* probe was fully retarded at both FleQ concentrations used (0.45 and 4.5 μM). As a negative control, a PCR fragment bearing the intergenic region of *P*. *putida* ORF PP1155, which was negative for FleQ regulation in our screening above, was digested with SacII and used as a probe. None of the two PP1155 promoter bands were substantially retarded, suggesting that our preparation of *P*.*putida* FleQ is and active capable of specific interaction with the *P*. *aeruginosa* P*cdrA* promoter region. Non-radioactive gel mobility shift assays were also performed using restriction enzyme-digested PCR products containing the P*lapA* and P*bcsD* promoter region fragments used for expression analysis above (Fig 8). At P*lapA*, 4.5 μM FleQ caused a full shift of the 550 and 297 bp bands, predicted to contain two and one FleQ binding sites, respectively. No alteration was observed in the mobility of the 152 bp band, not bearing any putative FleQ binding motifs. A similar result was obtained with the P*bcsD* promoter region: the 263 bp fragment, containing two putative sites, was fully retarded at 4.5 μM FleQ, while mobility of the additional 200 bp fragment was not altered. These results indicate that FleQ interacts specifically with the P*lapA* and P*bcsD* promoter regions. In addition, the high degree of correlation between the retarded fragments and the bioinformatics predictions is consistent with the notion that the predicted FleQ binding motifs indeed correspond to *bona fide* FleQ binding sites.

## Discussion

Our results provide evidence that FleQ is the master regulator of the flagellar cascade in *P*. *putida*. Phenotypic analyses revealed that a *fleQ* mutant is strongly defective in flagella-mediated swimming and swarming motility. On the other hand, gene expression analyses revealed seven FleQ-activated promoters within the flagellar biogenesis gene cluster, and two additional FleQ-activated promoters driving the synthesis of flagellar and chemotaxis proteins. The set of FleQ-activated motility-related promoters could be further divided into two subsets, three promoters directly activated by FleQ and strongly dependent on σ^N^, and six promoters indirectly activated by FleQ, and co-dependent on σ factors σ^N^ and FliA. The regulatory patterns in these two subsets are entirely consistent with those in Class II and Class IV flagellar promoters as defined in *P*. *aeruginosa* [10]. Even though our analysis is far from complete, we propose that a regulatory cascade reminiscent of (although arguably not identical to) that described in *P*. *aeruginosa* controls flagellar synthesis in *P*. *putida*. Additional evidence in this vein includes the conservation of the regulatory elements involved (FleQ, FleN, σ^N^, FleRS and FliA), and *in silico* analysis of multiple promoter regions in the flagellar cluster, showing the presence of putative σ^N^ or FliA binding sites (S4 Fig), consistent to the position of their *P*. *aeruginosa* counterparts as Class II, III or IV promoters in the flagellar cascade [73]. Flagellar gene regulation in *P*. *putida* was for the most part so far unexplored, and this study provides a fresh framework for further research on the regulation of the *P*. *putida* flagellar genes.

Our results also support the notion that c-di-GMP interferes with FleQ in the activation of the flagellar cascade. Increased c-di-GMP levels have been shown to negatively regulate flagellar function and flagellar biogenesis in multiple bacteria by a variety of mechanisms [74], and c-di-GMP has been shown to interact directly with *P*. *aeruginosa* FleQ to inhibit its ATPase activity, essential for σ^N^ promoter activation [13, 15]. This phenomenon is relevant to the transition from a motile to a sessile lifestyle, during which c-di-GMP stimulates the synthesis of biofilm matrix components [74] while preventing flagellar motility, which would otherwise contribute to destabilize the biofilm structure [75–77]. Four additional promoters driving the synthesis of proteins involved in c-di-GMP signaling (P*morA*, P*pp1144*, P*pp3711* and P*bifA*) showed a regulatory pattern consistent with those of Class IV flagellar promoters. Regulation of c-di-GMP signaling by the flagellar cascade was previously documented in *L*. *pneumophila* [78]. As c-di-GMP is an effector of FleQ, the regulation of these factors may operate as feedback loops to fine-tune FIeQ activity. It is interesting that two of these genes provide additional connections with the biofilm developmental cycle: MorA is a DGC that regulates both flagellar motility and biofilm development [36, 79], and BifA is a PDE responsible for the decrease of the c-di-GMP levels that trigger starvation-induced biofilm dispersal [35]. Regulation of BifA synthesis by the flagellar cascade is a clear-cut example of how a positive feedback loop may operate: decreased c-di-GMP levels induce the flagellar cascade, which in turn leads to FliA-dependent activation of BifA synthesis to decrease the c-di-GMP levels further, simultaneously stimulating flagellar synthesis and biofilm dispersal. This mechanism likely acts as a checkpoint to foster quick dispersal, provided that synthesis of functional flagella to faciltitate escape of the cells released from the biofilm is underway.

We also provide evidence of the involvement of FleQ as a major regulator of biofilm development. A *fleQ* mutant was strongly deficient in surface attachment and biofilm formation. FleQ was also responsible for mediating certain responses to elevated c-di-GMP levels, as a *fleQ* null mutation suppressed the aggregation, pellicle formation and CR adsorption phenotypes of a Δ*bifA* strain, displaying high intracellular c-di-GMP concentrations. This is in sharp contrast with the situation in *P*. *aeruginosa*, in which lack of FleQ leads to the formation of wrinkly colonies as well as overproduction of the Pel and Psl exopolysaccharides and the high molecular weight adhesin CdrA [10, 13]. However, a *P*. *fluorescens* Pf0-1 mutant lacking the FleQ ortholog AdnA was defective in adhesion and biofilm formation in a flagellum-independent fashion [80, 81], suggesting a regulatory scheme similar to that in *P*. *putida*, although AdnA regulation of biofilm matrix components has not yet been documented.

Two promoters driving the synthesis of important components of the biofilm matrix are targets for coordinate FleQ- and c-di-GMP-dependent regulation: P*lapA*, responsible for the synthesis of the high molecular weight adhesin LapA and P*bcsD*, directing the synthesis of the cellulose synthase complex. The direct involvement of FleQ in the regulation of *lapA* and *bcs* transcription was described very recently [39]. Our *In silico* analysis identified three and two putative FleQ binding sites at the P*lapA* and P*bcsD* promoter regions, respectively, and gel mobility shift analysis revealed that fragments containing these regions were specifically bound by FleQ. Therefore, our observations confirm and expand these results, providing evidence of the location of the FleQ binding elements.

Our results show that FleQ represses P*bcsD* transcription, and repression is prevented by high c-di-GMP levels. This regulatory pattern is qualitatively equivalent to that described recently by means of qRT-PCR using *bcsQ*-specific primers [39]. However, this work a greater extent of repression (~5-fold vs. ~1.5-fold) was observed, and our qRT-PCR results using *bcsD*-specific primers yielded even greater (>30-fold) repression. A second P*bcsD-gfp-lacZ* fusion including additional sequences (from -674 to +388), also yielded ~1.5-fold regulation (data not shown). The reasons for this discrepancy between the qRT-PCR and promoter fusion results are currently unknown, and may be be related to differences between the two experimenrtal systems, or to as of yet unrevealed regulatory features of the *bcs* cluster. FleQ repression that is antagonized by c-di-GMP is reminiscent of the regulation of the *pel*, *psl* and *cdrA* operons [13], encoding biofilm matrix components in *P*. *aeruginosa*. Stimulation of cellulose synthesis by c-di-GMP may also operate by a second mechanism. Inspection of the *P*. *putida* BcsA sequence revealed high conservation of the PilZ domain in the catalytic subunit BcsA. This domain has been shown to interact with c-di-GMP to induce cellulose synthase activity in other organisms [82–84], and therefore similar posttranscriptional regulation is likely to occur in *P*. *putida*.

Our results also show that FleQ activates P*lapA* transcription, and activation is stimulated by high c-di-GMP levels, again consistently with recently published results [39]. Synergistic activation of LapA synthesis by c-di-GMP and FleQ provide a rationale to the high pellicle production and aggregative phenotypes of a Δ*bifA* mutant and the suppression of these phenotypes by inactivation of *fleQ*. FleQ is a member of the well-characterized bacterial enhancer-binding protein (bEBP) family involved in activation of σ^N^-bearing RNA polymerase-dependent transcription. However, unlike the FleQ-activated flagellar promoters, P*lapA* is not σ^N^-dependent. Previously, FleQ has been shown to act as an activator of the σ^N^-independent promoters for the *P*. *aeruginosa pel* and *cdrA* operons in the presence of high c-di-GMP concentrations [17, 18]. A unique feature of P*lapA* is that it is activated by FleQ under both high and low c-di-GMP regimes, although c-di-GMP clearly stimulates activation. In contrast, *pel* and *cdrA* are repressed in the presence of low c-di-GMP levels, and activated when c-di-GMP levels are increased. Activation of σ^N^-dependent promoters by bEBPs involves a number of unique, highly specific mechanisms (binding to distant sites, oligomerization of the DNA-bound activator and ATP hydrolysis coupled to rearrangement of the transcription initiation complex) widely divergent from those observed in promoters dependent on other σ factors [85]. FleQ is unique among bEBPs in its ability to activate both σ^N^-dependent and σ^N^-independent transcription, and the elucidation of the mechanisms underlying the ability to interact with both forms of RNA polymerase is a main goal of our future research.

**Fig 9:**
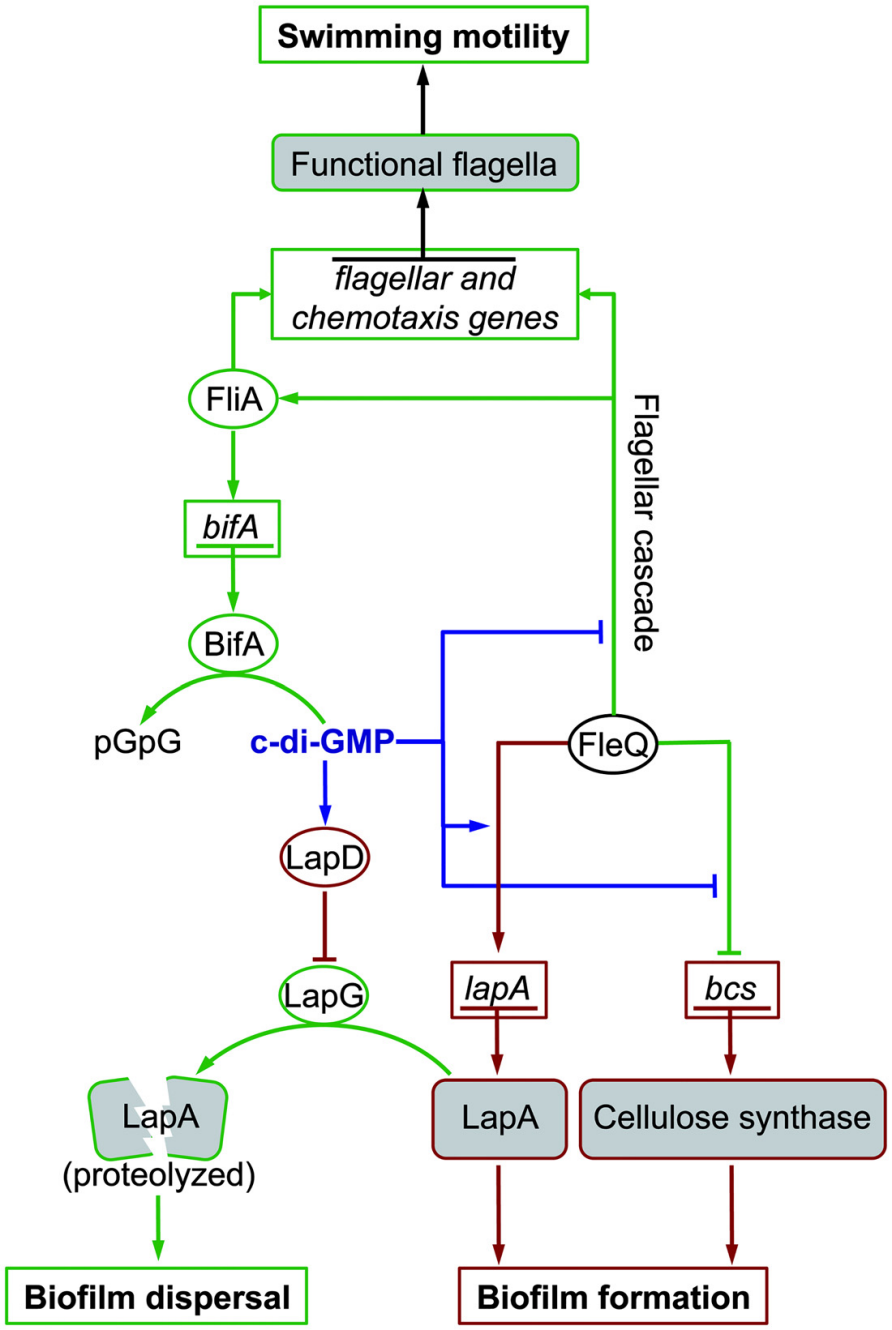
Working regulatory model. A simplified regulatory circuit for flagellar motility and biofilm development in *P*. *putida*. Genes are surrounded by boxes. Proteins are surrounded by ovals. Arrows denote positive effects. T-shaped line ends denote negative effects. The direct effect of c-di-GMP is indicated by blue lines. Processes and activities occurring preferentially in a low c-di-GMP regime are indicated by green lines. Processes and activities occurring preferentially in a high c-di-GMP regime are indicated by red lines.

Taken together, the results presented in this work can be integrated with the available knowledge in a working regulatory model in which c-di-GMP levels control the *P*. *putida* lifestyle switch by means of a complex regulatory network involving multiple regulatory and signal transduction elements (Fig 9). FleQ acts as a positive regulator of flagellar promoters, both directly, and indirectly, and its activity is antagonized by c-di-GMP. We believe it is safe to assume that a four-tiered flagellar cascade similar to that described in *P*. *aeruginosa* likely occurs in *P*. *putida* [10]. FleQ also indirectly activates genes encoding several putative c-di-GMP synthesizing and degrading proteins, which may generate regulatory feedback loops in the system. Finally, c-di-GMP positively regulates biofilm development by two separate mechanisms: firstly, by modulating the activity of FleQ to promote the synthesis of key components of the biofilm matrix, and secondly, by inhibiting the signal transduction circuit leading to starvation-induced dispersal [29, 30, 37]. While some of the details of this circuit need to be clarified, we believe it represents a valid framework for further research on the regulation of the lifestyle switch in *P*. *putida*.

## Supporting Information

S1 Additional References (PDF)

S1 Fig Complementation of the biofilm formation phenotype. Microtiter plate cultures of the wild-type KT2442 and the *fleQ* mutant bearing the empty miniTn*7*B-Gm transposon (Ø) or the miniTn*7*B-*fleQ* transposon expressing *fleQ* (+FleQ) were grown in LB and biofilm growth was assessed at 6 or 24 hours. Bars represent the averages and standard deviations of at least three independent experiments.
(PDF)

S2 Fig Swimming and swarming assays of the wild-type and *fleQ* strains. **Left.** Swimming assay, showing a picture of a typical swim plate with the wild-type (KT2442) and *fleQ* (MRB35) strains. **Right.** Swarming assay, showing a picture of a representative swarming plate with the wild-type (KT2442) and *fleQ* (MRB35) strains.
(PDF)

S3 Fig Purification of FleQ. SDS-PAGE showing FleQ overproduction and purification. Lane 1: uninduced cells. Lane 2: Induced cells. Lane 3: Clarified extract. Lane 4: Flow-through from affinity column. Lane 5: Wash from affinity column. Lane 6: resin sample prior to cleavage. Lane 7: flow-through from DTT addition. Lane 8: Eluted protein. Lane 9: resin sample after cleavage.
(PDF)

S4 Fig Sequence alignment of σ^N^- and FliA-dependent promoter regions. Aligned sequences of the FleQ-regulated σ^N^-dependent (top) and and FliA-dependent (bottom) promoter regions described in this work (black), and additional flagellar promoter regions present in the promoter library (red). Shaded positions indicate matches to the consensus. Consensus sequences of σ^N^ and FliA binding sites taken from [72] and [46], respectively.
(PDF)

S1 Table Promoters included in the ordered promoter library. ^1^ORF identification code according to the *Pseudomonas* genome annotation. ^2^Operon structure, according to the DOOR algorythm prediction for the *P*. *putida* KT2440 genome. ^3^Description of the relevant gene products and the criteria used for selection of each promoter.
(PDF)

S2 Table Results of the screenings for FleQ- and c-di-GMP-dependent regulation. The results shown are the averages and standard deviations of at least 3 independent experiments.
(PDF)

S3 Table Predicted FleQ binding sites at the P*lapA* and P*bcsD* promoter regions. Prediction was performed according to [18]. ^1^Coordinates are relative to the start codons. ^2^A plus (+) sign designates the strand corresponding to the *lapA* or *bcs* transcript ^3^Positions matching the consensus are indicated in bold.
(PDF)

## References

[1] Costerton JW, Lewandowski Z, Caldwell DE, Korber DR, Lappin-Scott HM. Microbial biofilms. Annu Rev Microbiol. 1995;49: 711–745. 8561477 10.1146/annurev.mi.49.100195.003431

[2] O'Toole GA, Kaplan HB, Kolter R. Biofilm formation as microbial development. Annu Rev Microbiol. 2000;54: 49–79. 11018124 10.1146/annurev.micro.54.1.49

[3] Monds RD, O'Toole GA. The developmental model of microbial biofilms: ten years of a paradigm up for review. Trends Microbiol. 2009;17: 73–87. 10.1016/j.tim.2008.11.001 19162483

[4] Hengge R. Principles of c-di-GMP signalling in bacteria. Nat Rev Microbiol 2009;7: 263–273. 10.1038/nrmicro2109 19287449

[5] Mills E, Pultz IS, Kulasekara HD, Miller SI. The bacterial second messenger c-di-GMP: mechanisms of signalling. Cell Microbiol. 2011;13: 1122–1129. 10.1111/j.1462-5822.2011.01619.x 21707905

[6] Boyd CD, O'Toole GA. Second messenger regulation of biofilm formation: breakthroughs in understanding c-di-GMP effector systems. Ann Rev Cell Dev Biol. 2012;28: 439–462. 23057745 10.1146/annurev-cellbio-101011-155705 PMC4936406

[7] Römling U. Cyclic di-GMP, an established secondary messenger still speeding up. Environ Microbiol. 2012;14: 1817–1829. 10.1111/j.1462-2920.2011.02617.x 22040037

[8] Römling U, Galperin MY, Gomelsky M. Cyclic di-GMP: the first 25 years of a universal bacterial second messenger. Microbiol Mol Biol Rev. 2013;77: 1–52. 10.1128/MMBR.00043-12 23471616 PMC3591986

[9] Arora SK, Ritchings BW, Almira EC, Lory S, Ramphal R. A transcriptional activator, FleQ, regulates mucin adhesion and flagellar gene expression in *Pseudomonas aeruginosa* in a cascade manner. J Bacteriol. 1997;179: 5574–5581. 9287015 10.1128/jb.179.17.5574-5581.1997 PMC179431

[10] Dasgupta N, Wolfgang MC, Goodman AL, Arora SK, Jyot J, Lory S, Ramphal R. A four-tiered transcriptional regulatory circuit controls flagellar biogenesis in *Pseudomonas aeruginosa*. Mol Microbiol. 2003;50: 809–824. 14617143 10.1046/j.1365-2958.2003.03740.x

[11] Smith TD, Hoover TR. Deciphering bacterial flagellar gene regulatory networks in the genomic era. Adv Appl Microbiol. 2009;67: 257–295. 10.1016/S0065-2164(08)01008-3 19245942

[12] Jyot J, Dasgupta N, Ramphal R. FleQ, the major flagellar gene regulator in *Pseudomonas aeruginosa*, binds to enhancer sites located either upstream or atypically downstream of the RpoN binding site. J Bacteriol. 2002;184: 5251–5260. 12218010 10.1128/JB.184.19.5251-5260.2002 PMC135358

[13] Hickman JW, Harwood CS. Identification of FleQ from *Pseudomonas aeruginosa* as a c-di-GMP-responsive transcription factor. Mol Microbiol. 2008;69: 376–389. 10.1111/j.1365-2958.2008.06281.x 18485075 PMC2612001

[14] Starkey M, Hickman JH, Ma L, Zhang N, De Long S, Hinz A, et al *Pseudomonas aeruginosa* rugose small-colony variants have adaptations that likely promote persistence in the cystic fibrosis lung. J Bacteriol. 2009;191: 3492–3503. 10.1128/JB.00119-09 19329647 PMC2681918

[15] Baraquet C, Harwood CS. Cyclic diguanosine monophosphate represses bacterial flagella synthesis by interacting with the Walker A motif of the enhancer-binding protein FleQ. Proc Natl Acad Sci USA. 2013;110: 18478–18483. 10.1073/pnas.1318972110 24167275 PMC3832005

[16] Matsuyama BY, Krasteva PV, Baraquet C, Harwood CS, Sondermann H, Navarro MV. Mechanistic insights into c-di-GMP-dependent control of the biofilm regulator FleQ from *Pseudomonas aeruginosa*. Proc Natl Acad Sci USA. 2016; 113: E209–E218. 10.1073/pnas.1523148113 26712005 PMC4720306

[17] Baraquet C, Murakami K, Parsek MR, Harwood CS. The FleQ protein from *Pseudomonas aeruginosa* functions as both a repressor and an activator to control gene expression from the *pel* operon promoter in response to c-di-GMP. Nucleic Acids Res. 2012;40: 7207–7218. 10.1093/nar/gks384 22581773 PMC3424551

[18] Baraquet C, Harwood CS. FleQ DNA binding consensus sequence revealed by studies of FleQ-dependent regulation of biofilm gene expression in *Pseudomonas aeruginosa*. J Bacteriol. 2016;198: 178–186. 26483521 10.1128/JB.00539-15 PMC4686206

[19] Dasgupta N, Arora SK, Ramphal R. *fleN*, a gene that regulates flagellar number in *Pseudomonas aeruginosa*. J Bacteriol. 2000;182: 357–364. 10629180 10.1128/jb.182.2.357-364.2000 PMC94283

[20] Dasgupta N, Ramphal R. Interaction of the antiactivator FleN with the transcriptional activator FleQ regulates flagellar number in *Pseudomonas aeruginosa*. J Bacteriol. 2001;183: 6636–6644. 11673434 10.1128/JB.183.22.6636-6644.2001 PMC95495

[21] Martins dos Santos VAP, Heim S, Moore ERB, Strätz M, Timmis KN. Insights into the genomic basis of niche specificity of *Pseudomonas putida*. Environ Microbiol. 2004;6: 1264–1286. 15560824 10.1111/j.1462-2920.2004.00734.x

[22] Klausen M, Gjermansen M, Kreft JU, Tolker-Nielsen T. Dynamics of development and dispersal in sessile microbial communities: examples from *Pseudomonas aeruginosa* and *Pseudomonas putida* model biofilms. FEMS Microbiol Lett. 2006;261: 1–11. 16842351 10.1111/j.1574-6968.2006.00280.x

[23] Martínez-Gil, Ramos-González MI, Espinosa-Urgel M. Roles of cyclic di-GMP and the Gac system in transcriptional control of the genes coding for the *Pseudomonas putida* adhesins LapA and LapF. J Bacteriol. 2014;196: 1484–1495. 10.1128/JB.01287-13 24488315 PMC3993364

[24] Steinberger RE, Holden PA. Extracellular DNA in single- and multiple-species unsaturated biofilms. Appl Environ Microbiol. 2005;71: 5404–5410. 16151131 10.1128/AEM.71.9.5404-5410.2005 PMC1214645

[25] Camesano TA, and Abu-Lail NI. Heterogeneity in bacterial surface polysaccharides, probed on a single-molecule basis. Biomacromolecules. 2002;3: 661–667. 12099808 10.1021/bm015648y

[26] Chang W-S, Halverson LJ. Reduced water availability influences the dynamics, development, and ultrastructural properties of *Pseudomonas putida* biofilms. J Bacteriol. 2003;185: 6199–6204. 14526033 10.1128/JB.185.20.6199-6204.2003 PMC225025

[27] Chang W-S, van de Mortel M, Nielsen L, Nino de Guzmán G, Li X, Halverson LJ. Alginate production by *Pseudomonas putida* creates a hydrated microenvironment and contributes to biofilm architecture and stress tolerance under water-limiting conditions. J Bacteriol. 2007;189: 8290–8299. 17601783 10.1128/JB.00727-07 PMC2168710

[28] Nilsson M, Chiang W-C, Fazli M, Gjermansen M, Givskov M, Tolker-Nielsen T. Influence of putative exopolysaccharide genes on *Pseudomonas putida* KT2440 biofilm stability. Environ Microbiol. 2011;13: 1357–1369. 10.1111/j.1462-2920.2011.02447.x 21507178

[29] Gjermansen M, Ragas P, Sternberg C, Molin S, Tolker-Nielsen T. Characterization of starvation-induced dispersion in *Pseudomonas putida* biofilms. Environ Microbiol. 2005;7: 894–904. 15892708 10.1111/j.1462-2920.2005.00775.x

[30] Gjermansen M, Nilsson M, Yang L, Tolker-Nielsen T. Characterization of starvation-induced dispersion in *Pseudomonas putida* biofilms: genetic elements and molecular mechanisms. Mol Microbiol. 2010;75: 815–826. 10.1111/j.1365-2958.2009.06793.x 19602146

[31] Newell PD, Monds RD, O’Toole GA. LapD is a bis-(3',5')-cyclic dimeric GMP-binding protein that regulates surface attachment by *Pseudomonas fluorescens* Pf0-1. Proc Natl Acad Sci USA. 2009;106: 3461–3466. 10.1073/pnas.0808933106 19218451 PMC2651287

[32] Matilla MA, Travieso ML, Ramos JL, Ramos-González MI. Cyclic diguanylate turnover mediated by the sole GGDEF/EAL response regulator in *Pseudomonas putida*: its role in the rhizosphere and an analysis of its target processes. Environ Microbiol. 2011;13: 1745–1766. 10.1111/j.1462-2920.2011.02499.x 21554519

[33] Gjermansen M, Ragas P, Tolker-Nielsen T. Proteins with GGDEF and EAL domains regulate *Pseudomonas putida* biofilm formation and dispersal. FEMS Microbiol Lett. 2006;265: 215–224. 17054717 10.1111/j.1574-6968.2006.00493.x

[34] Galperin MY. A census of membrane-bound and intracellular signal transduction proteins in bacteria: bacterial IQ, extroverts and introverts. BMC Microbiol. 2005;5: 35 15955239 10.1186/1471-2180-5-35 PMC1183210

[35] Ulrich LE, Zhulin IB. MiST: a microbial signal transduction database. Nucleic Acids Res. 2007;35: D386–D390. 17135192 10.1093/nar/gkl932 PMC1747179

[36] Choy WK, Zhou L, Syn CK, Zhang LH, Swarup S. MorA defines a new class of regulators affecting flagellar development and biofilm formation in diverse *Pseudomonas* species. J Bacteriol. 2004;186: 7221–7228. 15489433 10.1128/JB.186.21.7221-7228.2004 PMC523210

[37] Jiménez-Fernández A, López-Sánchez A, Calero P, Govantes F. The c-di-GMP phosphodiesterase BifA regulates biofilm development in *Pseudomonas putida*. Environ Microbiol Reports. 2015;7: 78–84. 10.1111/1758-2229.12153 25870874

[38] Martínez-Gil M, Yousef-Coronado F, Espinosa-Urgel M. LapF, the second largest *Pseudomonas putida* protein, contributes to plant root colonization and determines biofilm architecture. Mol Microbiol. 2010;77: 549–561. 10.1111/j.1365-2958.2010.07249.x 20545856

[39] Xiao Y, Nie H, Liu H, Luo X, Chen W, Huang Q. C-di-GMP regulates the expression of *lapA* and *bcs* operons via FleQ in *Pseudomonas putida* KT2440. Environ Microbiol Rep. 2016 In press. 10.1111/1758-2229.12419 27120564

[40] López-Sánchez A, Leal-Morales A, Jiménez-Díaz L, Platero AI, Bardallo-Pérez J, Díaz-Romero A, et al Biofilm formation-defective mutants in *Pseudomonas putida*. FEMS Microbiol Lett. 2016;363: pii: fnw127 10.1093/femsle/fnw127 27190143

[41] Sambrook J, Russell DW, Russell D. Molecular cloning, a laboratory manual. Cold Spring Harbor, New York, USA: Cold Spring Harbor Laboratory Press; 2000

[42] Hanahan D. Studies on transformation of *Escherichia coli* with plasmids. J Mol Biol 1983;166: 557–580. 6345791 10.1016/s0022-2836(83)80284-8

[43] Govantes F, Molina-López JA, Santero E. Mechanism of coordinated synthesis of the antagonistic regulatory proteins NifL and NifA of *Klebsiella pneumoniae*. J Bacteriol. 1996; 178:6817–6823. 8955302 10.1128/jb.178.23.6817-6823.1996 PMC178581

[44] Franklin FC, Bagdasarian M, Bagdasarian MM, Timmis KN. Molecular and functional analysis of the TOL plasmid pWWO from *Pseudomonas putida* and cloning of genes for the entire regulated aromatic ring *meta* cleavage pathway. Proc Natl Acad Sci USA. 1981;78: 7458–7462. 6950388 10.1073/pnas.78.12.7458 PMC349287

[45] Köhler T, Harayama S, Ramos JL, Timmis KN. Involvement of *Pseudomonas putida* RpoN sigma factor in regulation of various metabolic functions. J Bacteriol. 1989;171: 4326–4333. 2666396 10.1128/jb.171.8.4326-4333.1989 PMC210208

[46] Rodríguez-Herva JJ, Duque E, Molina-Henares MA, Navarro-Avilés G, van Dilleijn P, de la Torre J, et al Physiological and transcriptomic characterization of a *fliA* mutant of *Pseudomonas putida* KT2440. Environ Microbiol Rep. 2010;2: 373–380. 10.1111/j.1758-2229.2009.00084.x 23766109

[47] López-Sánchez A, Jiménez-Fernández A, Calero P, Gallego LD, Govantes F. New methods for the isolation and characterization of biofilm-persistent mutants in *Pseudomonas putida*. Environ Microbiol Rep. 2013;5: 679–685. 10.1111/1758-2229.12067 24115618

[48] Porrúa O, García-González V, Santero E, Shingler V, Govantes F. Activation and repression of a σ^N^-dependent promoter naturally lacking upstream activation sequences. Mol Microbiol. 2009;73: 419–433. 10.1111/j.1365-2958.2009.06779.x 19570137

[49] Figurski DH, Helinski DR. Replication of an origin-containing derivative of plasmid RK2 dependent on a plasmid function provided in *trans*. Proc Natl Acad Sci USA. 1979;76: 1648–1652. 377280 10.1073/pnas.76.4.1648 PMC383447

[50] Shetty RP, Endy D, Knight TF Jr. Engineering BioBrick vectors from BioBrick parts. J Biol Eng. 2008;2: 5 10.1186/1754-1611-2-5 18410688 PMC2373286

[51] Choi KH, Gaynor JB White KG, Lopez C, Bosio CM, Karkhoff-Schweizer RR, Schweizer HP. A Tn7-based broad-range bacterial cloning and expression system. Nat Methods. 2005;2: 443–448. 15908923 10.1038/nmeth765

[52] Ausmees N, Mayer R, Weinhouse H, Volman G, Amikam D, Benziman M, Lindberg M. Genetic data indicate that proteins containing the GGDEF domain possess diguanylate cyclase activity. FEMS Microbiol Lett. 2001;204: 163–167. 11682196 10.1111/j.1574-6968.2001.tb10880.x

[53] O’Toole GA, Pratt LA, Watnick PI, Newman DK, Weaver VB, Kolter R. Genetic approaches to study of biofilms. Methods Enzymol. 1999;310: 91–109. 10547784 10.1016/s0076-6879(99)10008-9

[54] An S, Wu J, Zhang L-H. Modulation of *Pseudomonas aeruginosa* biofilm dispersal by a cyclic-di-GMP phosphodiesterase with a putative hypoxia-sensing domain. Appl Environ Microbiol. 2010;76: 8160–8173. 10.1128/AEM.01233-10 20971871 PMC3008239

[55] Espinosa-Urgel M, Salido A, Ramos JL. Genetic analysis of functions involved in adhesion of *Pseudomonas putida* to seeds. J Bacteriol. 2000;182: 2363–2369. 10762233 10.1128/jb.182.9.2363-2369.2000 PMC111295

[56] Choi KH, Kumar A, Schweizer HP. A 10-min method for preparation of highly electrocompetent *Pseudomonas aeruginosa* cells: application for DNA fragment transfer between chromosomes and plasmid transformation. J Microbiol Methods. 2006;64: 391–397. 15987659 10.1016/j.mimet.2005.06.001

[57] Hoang TT, Karkhoff-Schweizer RR, Kutchma AJ, Schweizer HP. A broad-host-range Flp-FRT recombination system for site-specific excision of chromosomally located DNA sequences: application for isolation of unmarked *Pseudomonas aeruginosa* mutants. Gene. 1998;212: 77–86. 9661666 10.1016/s0378-1119(98)00130-9

[58] Llamas MA, Ramos JL, Rodríguez-Herva JJ. Mutations in each of the *tol* genes of *Pseudomonas putida* reveal that they are critical for maintenance of outer membrane stability. J Bacteriol. 2000;182: 4764–4772. 10940016 10.1128/jb.182.17.4764-4772.2000 PMC111352

[59] Nelson KE, Weinel C, Paulsen IT, Dodson RJ, Hilbert H, Martins dos Santos VA, et al Complete genome sequence and comparative analysis of the metabolically versatile *Pseudomonas putida* KT2440. Environ Microbiol. 2002; 4:799–808. 12534463 10.1046/j.1462-2920.2002.00366.x

[60] Mao F, Dam P, Chou J, Olman V, Xu Y. DOOR: a database for prokaryotic operons. Nucleic Acids Res. 2009;37: D459–463. 10.1093/nar/gkn757 18988623 PMC2686520

[61] Brouwer RW, Kuipers OP, van Hijum SA. The relative value of operon predictions. Brief Bioinform. 2008;9: 367–375. 10.1093/bib/bbn019 18420711

[62] Parkinson JS. *cheA*, *cheB*, and *cheC* genes of *Escherichia coli* and their role in chemotaxis. J Bacteriol. 1976;126: 758–770. 770453 10.1128/jb.126.2.758-770.1976 PMC233211

[63] Matilla MA, Ramos JL, Duque E, de Dios Alché J, Espinosa-Urgel M, Ramos-González MI. Temperature and pyoverdine-mediated iron acquisition control surface motility of *Pseudomonas putida*. Environ Microbiol. 2007;9: 1842–50. 17564617 10.1111/j.1462-2920.2007.01286.x

[64] Miller JH. A short course in bacterial genetics: a laboratory manual. Cold Spring Harbor, New York: Cold Spring Harbor Laboratory Press; 1992.

[65] García-González V, Govantes F, Porrúa O, Santero E. Regulation of the *Pseudomonas* sp. strain ADP cyanuric acid degradation operon. J Bacteriol. 2005;187: 155–167. 15601699 10.1128/JB.187.1.155-167.2005 PMC538813

[66] Yuste L, Hervás AB, Canosa I, Tobes R, Jiménez JI, Nogales J, et al Growth phase-dependent expression of the *Pseudomonas putida* KT2440 transcriptional machinery analysed with a genome-wide DNA microarray. Environ Microbiol. 2006; 8: 165–177. 16343331 10.1111/j.1462-2920.2005.00890.x

[67] Yang CH, Menge JA, Cooksey DA. Mutations affecting hyphal colonization and pyoverdine production in pseudomonads antagonistic toward *Phytophthora parasitica*. Appl Environ Microbiol. 1994;60: 473–481.matines 16349177 10.1128/aem.60.2.473-481.1994 PMC201336

[68] Turnbull GA, Morgan JA, Whipps JM, Saunders JR. The role of motility in the *in vitro* attachment of *Pseudomonas putida* PaW8 to wheat roots. FEMS Microbiol Ecol. 2001;35: 57–65. 11248390 10.1111/j.1574-6941.2001.tb00788.x

[69] Yousef-Coronado F, Travieso ML, Espinosa-Urgel M. Different, overlapping mechanisms for colonization of abiotic and plant surfaces by *Pseudomonas putida*. FEMS Microbiol Lett. 2008;288: 118–124. 10.1111/j.1574-6968.2008.01339.x 18783437

[70] Reyes-Darias JA, García V, Rico-Jiménez M, Corral-Lugo A, Lesouhaitier O, Juárez-Hernández D, et al Specific gamma-aminobutyrate chemotaxis in pseudomonads with different lifestyle. Mol Microbiol. 2015;97: 488–501. 10.1111/mmi.13045 25921834

[71] Parkins MD, Ceri H, Storey DG. *Pseudomonas aeruginosa* GacA, a factor in multihost virulence, is also essential for biofilm formation. Mol Microbiol. 2001;40: 1215–1226. 11401724 10.1046/j.1365-2958.2001.02469.x

[72] Grant CE, Bailey TL, Noble WS. FIMO: Scanning for occurrences of a given motif. Bioinformatics. 2011;27: 1017–1018. 10.1093/bioinformatics/btr064 21330290 PMC3065696

[73] Cases I, Ussery DW, de Lorenzo V. The sigma54 regulon (sigmulon) of *Pseudomonas putida*. Environ Microbiol. 2003;5: 1281–1293. 14641574 10.1111/j.1462-2920.2003.00528.x

[74] Guttenplan S, Kearns DB. Regulation of flagellar motility during biofilm formation. Microbiol Rev. 2013;37: 849–871. 10.1111/1574-6976.12018 PMC3718880 23480406

[75] O’Toole GA, Kolter R. Flagellar and twitching motility are necessary for *Pseudomonas aeruginosa* biofilm development. Mol Microbiol. 1998;30: 295–304. 9791175 10.1046/j.1365-2958.1998.01062.x

[76] Sauer K, Camper AK, Ehrlich GD, Costerton JW, Davies DG. *Pseudomonas aeruginosa* displays multiple phenotypes during development as a biofilm. J Bacteriol. 2002;184: 1140–1154. 11807075 10.1128/jb.184.4.1140-1154.2002 PMC134825

[77] Klausen M, Heydorn A, Ragas P, Lambertsen L, Aaes-Jørgensen A, Molin S, Tolker-Nielsen T. Biofilm formation by *Pseudomonas aeruginosa* wild type, flagella, and type IV pili mutants. Mol Microbiol. 2003;48: 1511–1524. 12791135 10.1046/j.1365-2958.2003.03525.x

[78] Albert-Weissenberger C, Sahr T, Sismeiro O, Hacker J, Heuner K, Buchrieser C. Control of flagellar gene regulation in Legionella pneumophila and its relation to growth phase. J Bacteriol. 2010;192: 446–55 10.1128/JB.00610-09 19915024 PMC2805308

[79] Meissner A, Wild V, Simm R, Rohde M, Erck C, Bredenbruch F, et al *Pseudomonas aeruginosa cupA*-encoded fimbriae expression is regulated by a GGDEF and EAL domain-dependent modulation of the intracellular level of cyclic diguanylate. Environ Microbiol. 2007;9: 2475–2485. 17803773 10.1111/j.1462-2920.2007.01366.x

[80] Casaz P, Happel A, Keithan J, Read DL, Strain SR, Levy SB. The *Pseudomonas fluorescens* transcription activator AdnA is required for adhesion and motility. Microbiology. 2001;147: 355–361. 11158352 10.1099/00221287-147-2-355

[81] Robleto EA, Lopez-Hernandez I, Silby MW, Levy SB. Genetic analysis of the AdnA regulon in *Pseudomonas fluorescens*: nonessential role of flagella in adhesion to sand and biofilm formation. J Bacteriol. 2003;185: 453–460. 12511490 10.1128/JB.185.2.453-460.2003 PMC145307

[82] Ross P, Weinhouse H, Aloni Y, Michaeli D, Weinberger-Ohana P, Mayer R, et al Regulation of cellulose synthesis in *Acetobacter xylinum* by cyclic diguanylic acid. Nature 1987;325: 279–281. 18990795 10.1038/325279a0

[83] Ryjenkov DA, Simm R, Römling U, Gomelsky M. The PilZ domain is a receptor for the second messenger c-di-GMP: the PilZ domain protein YcgR controls motility in enterobacteria. J Biol Chem. 2006;281: 30310–30314. 16920715 10.1074/jbc.C600179200

[84] Fang X, Ahmad I, Blanka A, Schottkowski M, Cimdins A, Galperin MY, et al GIL, a new c-di-GMP-binding protein domain involved in regulation of cellulose synthesis in enterobacteria. Mol Microbiol. 2014;93: 439–452. 10.1111/mmi.12672 24942809 PMC4116459

[85] Joly N, Zhang N, Buck M, Zhang X. Coupling AAA protein function to regulated gene expression. Biochim Biophys Acta. 2012;1823: 108–116. 10.1016/j.bbamcr.2011.08.012 21906631

