# Supplementary material for: Complex Interplay between FleQ, Cyclic Diguanylate and Multiple σ Factors Coordinately Regulates Flagellar Motility and Biofilm Development in Pseudomonas putida
Source: PLoS One. 2016 Sep 16;11(9):e0163142. doi: 10.1371/journal.pone.0163142 (PMC5026340; doi:10.1371/journal.pone.0163142)
Supplement: S1 Additional References — (PDF) [file pone.0163142.s001.pdf]

S1 Additional references

1S. Hinsa SM, Espinosa-Urgel M, Ramos JL, O'Toole GA. Transition from reversible to irreversible attachment during biofilm formation by *Pseudomonas* fluorescens WCS365 requires an ABC transporter and a large secreted protein. Mol Microbiol. 2003; 49: 905-918.

2S. Wagner VE, Bushnell D, Passador L, Brooks AI, Iglewski BH. Microarray analysis of *Pseudomonas aeruginosa* quorum-sensing regulons: effects of growth phase and environment. J Bacteriol. 2013; 185: 2080-2095.

3S. Fernández-Piñar R, Ramos JL, Rodríguez-Herva JJ, Espinosa-Urgel M. A two-component regulatory system integrates redox state and population density sensing in *Pseudomonas putida*. *J. Bacteriol*. 2008; 190: 7666-7674.

4S. Tolker-Nielsen T, Molin S. The biofilm lifestyle of Pseudomonads. In: Ramos JL, editor. *Pseudomonas,* Vol. 1. New York: Kluwer Academic/Plenum Press; 2004. pp. 547-571

5S. Srivastava S, Yadav A, Seem K, Mishra S, Chaudhary V, Nautiyal CS. Effect of high temperature on *Pseudomonas putida* NBRI0987 biofilm formation and expression of stress sigma factor RpoS. Curr Microbiol. 2008; 56: 453-457.

6S. Molina MA, Ramos JL, Espinosa-Urgel M. A two-partner secretion system is involved in seed and root colonization and iron uptake by *Pseudomonas putida* KT2440. Environ Microbiol 2006; 8: 639-647.

7S. Sauer K, Camper AK. Characterization of phenotypic changes in *Pseudomonas putida* in response to surface-associated growth. J. Bacteriol. 2001; 183: 6579-6589.

8S. van de Mortel M, Halverson LJ. Cell envelope components contributing to biofilm growth and survival of *Pseudomonas putida* in low-water-content habitats. 2004; Mol Microbiol. 52: 735-750.

9S. Espinosa-Urgel M, Ramos JL. Cell density-dependent gene contributes to efficient seed colonization by *Pseudomonas putida* KT2440. Appl Environ Microbiol. 2004; 70: 5190-5198.

10S. Hansen SK, Haagensen JA, Gjermansen M, Jørgensen TM, Tolker-Nielsen T, Molin S. Characterization of a *Pseudomonas putida* rough variant evolved in a mixed-species biofilm with Acinetobacter sp. strain C6. J Bacteriol. 2007; 189: 4932-4943.
