# Supplementary material for: Complex Interplay between FleQ, Cyclic Diguanylate and Multiple σ Factors Coordinately Regulates Flagellar Motility and Biofilm Development in Pseudomonas putida
Source: PLoS One. 2016 Sep 16;11(9):e0163142. doi: 10.1371/journal.pone.0163142 (PMC5026340; doi:10.1371/journal.pone.0163142)

S1 Figure. Complementation of the biofilm formation phenotype. Microtiter plate cultures of the wild-type KT2442 and the *fleQ*⁻ mutant bearing the empty miniTn*7*BB-Gm transposon (Ø) or the miniTn*7*BB-*fleQ* transposon expressing *fleQ* (+FleQ) were grown in LB and biofilm growth was assessed at 6 or 24 hours. Bars represent the averages and standard deviations of at least three independent experiments.

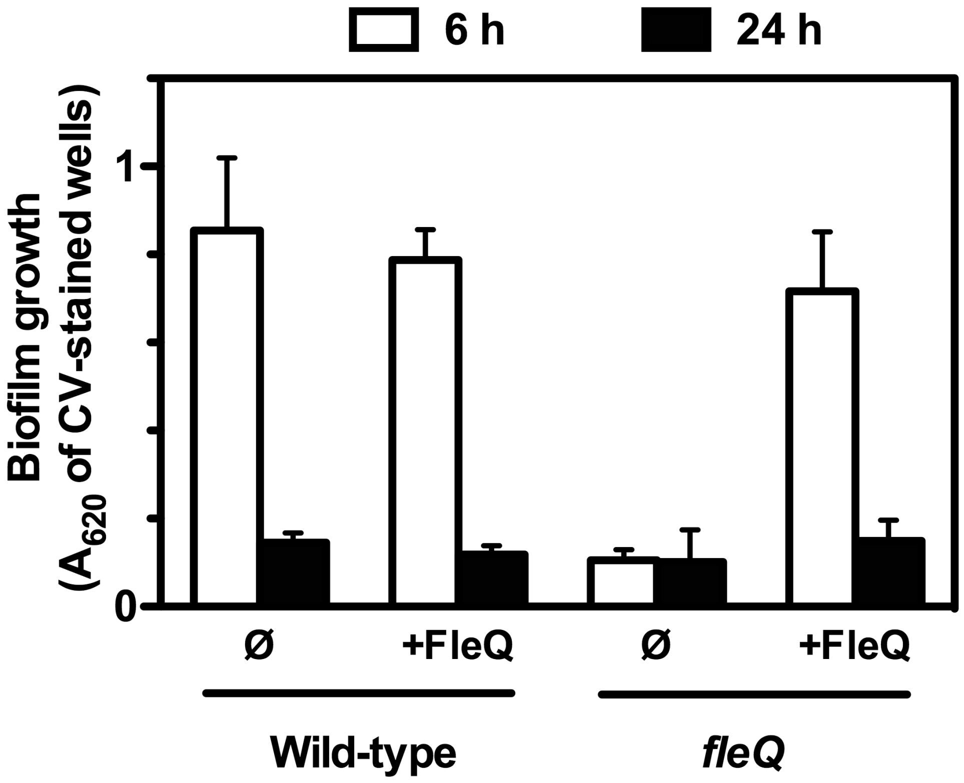

Supplement: S1 Fig — Microtiter plate cultures of the wild-type KT2442 and the fleQ mutant bearing the empty miniTn7B-Gm transposon (Ø) or the miniTn7B-fleQ transposon expressing fleQ (+FleQ) were grown in LB and biofilm growth was assessed at 6 or 24 hours. Bars represent the averages and standard deviations of at least three independent experiments. (PDF) [file pone.0163142.s002.pdf]
