# Supplementary material for: Complex Interplay between FleQ, Cyclic Diguanylate and Multiple σ Factors Coordinately Regulates Flagellar Motility and Biofilm Development in Pseudomonas putida
Source: PLoS One. 2016 Sep 16;11(9):e0163142. doi: 10.1371/journal.pone.0163142 (PMC5026340; doi:10.1371/journal.pone.0163142)

S2 Figure. Swimming and swarming assays of the wild-type and *fleQ*$^{-}$ strains. Left. Swimming assay, showing a picture of a typical swim plate with the wild-type (KT2442) and *fleQ*$^{-}$ (MRB35) strains. **Right.** Swarming assay, showing a picture of a representative swarming plate with the wild-type (KT2442) and *fleQ*$^{-}$ (MRB35) strains.

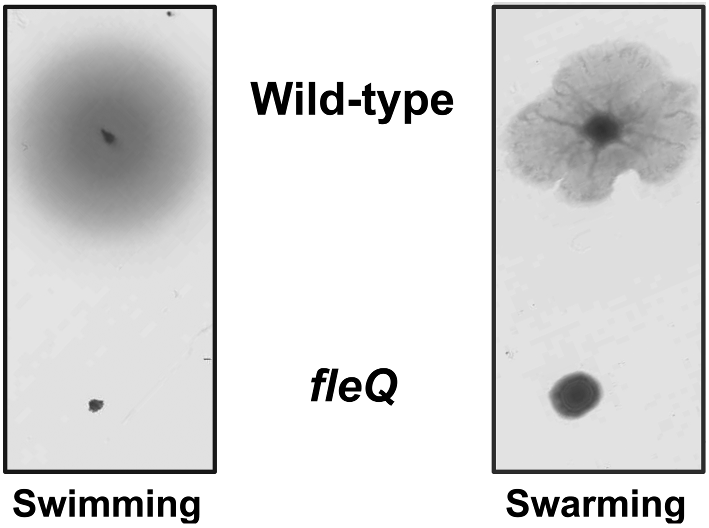

Supplement: S2 Fig — Left. Swimming assay, showing a picture of a typical swim plate with the wild-type (KT2442) and fleQ (MRB35) strains. Right. Swarming assay, showing a picture of a representative swarming plate with the wild-type (KT2442) and fleQ (MRB35) strains. (PDF) [file pone.0163142.s003.pdf]
