# Supplementary material for: Complex Interplay between FleQ, Cyclic Diguanylate and Multiple σ Factors Coordinately Regulates Flagellar Motility and Biofilm Development in Pseudomonas putida
Source: PLoS One. 2016 Sep 16;11(9):e0163142. doi: 10.1371/journal.pone.0163142 (PMC5026340; doi:10.1371/journal.pone.0163142)

S3 Figure. Purification of FleQ. Coomassie blue-stained SDS-PAGE showing FleQ overproduction and purification. Lane M: size marker (molecular weight in kDa). Lane 1: uninduced cells. Lane 2: Induced cells. Lane 3: Clarified extract. Lane 4: Flow-through from chitin affinity column. Lane 5: Wash from affinity column. Lane 6: chitin resin sample prior to intein cleavage. Lane 7: flow-through from DTT addition. Lane 8: Eluted protein. Lane 9: chitin resin sample after intein cleavage. Arrows indicate the location of the fusion protein (CBD-Int-FleQ) and the cleavage products (CBD-Int and FleQ)

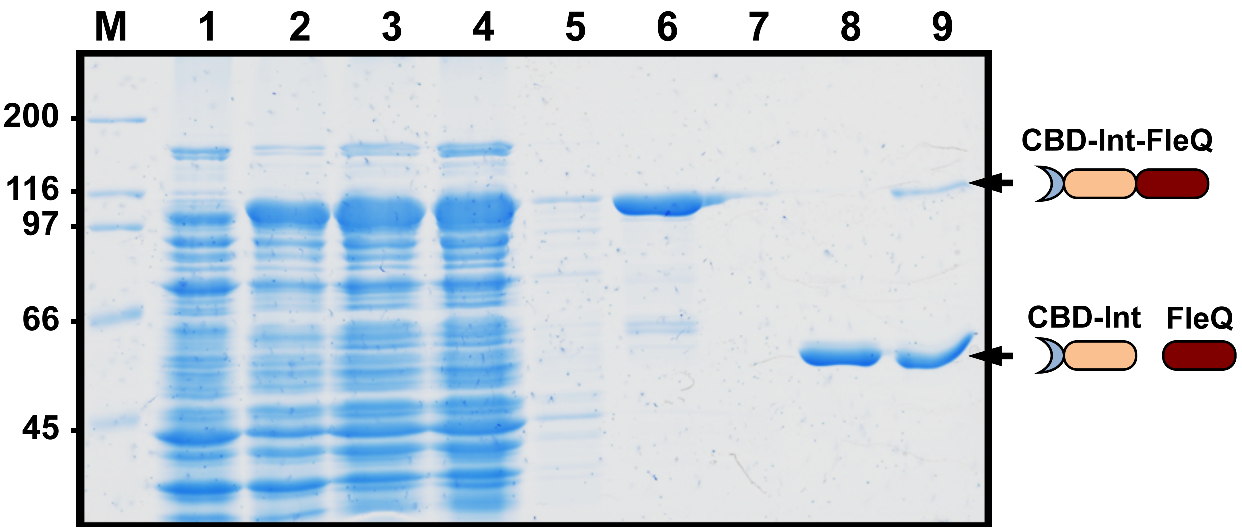

Supplement: S3 Fig — SDS-PAGE showing FleQ overproduction and purification. Lane 1: uninduced cells. Lane 2: Induced cells. Lane 3: Clarified extract. Lane 4: Flow-through from affinity column. Lane 5: Wash from affinity column. Lane 6: resin sample prior to cleavage. Lane 7: flow-through from DTT addition. Lane 8: Eluted protein. Lane 9: resin sample after cleavage. (PDF) [file pone.0163142.s004.pdf]
