# Supplementary material for: Complex Interplay between FleQ, Cyclic Diguanylate and Multiple σ Factors Coordinately Regulates Flagellar Motility and Biofilm Development in Pseudomonas putida
Source: PLoS One. 2016 Sep 16;11(9):e0163142. doi: 10.1371/journal.pone.0163142 (PMC5026340; doi:10.1371/journal.pone.0163142)
Supplement: S4 Fig — Aligned sequences of the FleQ-regulated σN-dependent (top) and and FliA-dependent (bottom) promoter regions described in this work (black), and additional flagellar promoter regions present in the promoter library (red). Shaded positions indicate matches to the consensus. Consensus sequences of σN and FliA binding sites taken from [72] and [46], respectively. (PDF) [file pone.0163142.s005.pdf]

S4 Figure. Sequence alignment of σ[N]- and FliA-dependent promoter regions. Aligned sequences of the FleQ-regulated σ[N]-dependent (top) and and FliA-dependent (bottom) promoter regions described in this work (black), and additional flagellar promoter regions present in the promoter library (red). Shaded positions indicate matches to the consensus. Consensus sequences of σ[N] and FliA binding sites taken from [72] and [46], respectively.

σ[N]-dependent promoters

```
PflhA       ATCTTTTCGTGTGGGTGTCTGCCAAAGTTGGAAAGCTTCTTGCAAAGCCACG
PflgB       ACCCCATAAATACGGGCTTTCCAGTGGTTGGCACAGCCCTTGCTATGCCTTG
PflgA       GCCTTTCGTTGCAACTCAGGTAGCGAGTCGGCACGGGCTTTGCTTTTTTGAG
PfleS       TCCTACGTCAAACCCAAACCCCCATCTCCGGCACGGCTATTGCTACACCGCT
PfliE       TGATCTCTGGGAAATGAGTTGCGAAGGCTGGCACCTTTGTTGCTTTAGGTAG
PfliD       ATGTGTAATGTTGGCATGAGTCTTGACTCGGATTGTGTCTCGCTCATTCATC
PfliK       ACGTATTTCCGCAGGCTGATACGCGAGTTGGCGCGAGTCTTGCCTGTCTCTG
PflgF       AAGCCCCGTAAACCGGGTGTTTCAGACTTGGTTCAATTATTGCTTGGAACCT
Consensus                                   TGGCAC N5   TTGCT
```

FliA-dependent promoters

```
PmcpG       CTCTTTAATCCTCTGCTCAAGCTTTAGCT TAATTCGGCCGATACCTGTGGG
PfliS       CGACTGATGGTCGTATTCAAGATCTTTGG  GGCTAAGCCGATCACTTAGGT
PfliC       TTTTTTGAAAAAACCCTCAAGCAACCCGC  GCACCCGACGATAACCATTAC
PflgM       GCAATGCATTTGTGCCTAAAGTTTATATC  GGGTTGGCCGAAAACAAGGCA
PcheV3      TTCGTGTGCATGACACTCAAAAAAGCAAA  GCCCGTGCCGACTCGCTACCT
Ppp4328     CCAGAACCCTTGTGTCTCAAGTCGCCCCG  CGCGCTGCCGTTATACTGGCA
Ppp3711     ATGCCACCATCGCTTTCCAAGACCCGGCC GCCGCTTGCCGGGGTCCCTTTT
PmorA       GGGCAGCCACCATCGGTCAACATGGCTGG   CACTTCGCCATAATCCGAGG
PbifA       GATTGATACAGCGCATTCAAGTTGCGGGG TCAGGCAACCGATACACTGCTA
Ppp1144     CATTAGTCAGGAAGGCTTAAGTACTCCTTCCTATACTGCCTCTGGACAGTCT
PcheY       GACGAAGAGCTGAACCTCAAGGAAATCGG  TGAGGTGCTGGGTGTCAGCGA
Consensus       T           TCAAG       N14-15   GCCGATA C
```
