# Supplementary material for: Complex Interplay between FleQ, Cyclic Diguanylate and Multiple σ Factors Coordinately Regulates Flagellar Motility and Biofilm Development in Pseudomonas putida
Source: PLoS One. 2016 Sep 16;11(9):e0163142. doi: 10.1371/journal.pone.0163142 (PMC5026340; doi:10.1371/journal.pone.0163142)
Supplement: S1 Table — 1ORF identification code according to the Pseudomonas genome annotation. 2Operon structure, according to the DOOR algorythm prediction for the P. putida KT2440 genome. 3Description of the relevant gene products and the criteria used for selection of each promoter. (PDF) [file pone.0163142.s006.pdf]

S1 Table. Promoters included in the ordered promoter library. [1]ORF identification code according to the *Pseudomonas* genome annotation. [2]Operon structure, according to the DOOR algorythm prediction for the *P. puitda* KT2440 genome. [3]Description of the relevant gene products and the criteria used for selection of each promoter.

ORF ID[1]	Operon prediction[2]	Description/selection criteria[3]
PP0127	*pp0127-pp0128-pp0129*	*pp0129* encodes a GGDEF domain protein.
PP0131	*pp0131*	Predicted signal transduction protein containing sensor and EAL domains.
PP0133	*algB-pp0132*	AlgB alginate biosynthesis Fis family transcriptional regulator.
PP0164	*lapGD*	LapG protease and LapD a c-di-GMP sensor, both involved in biofilm dispersal [27, 28].
PP0167	*lapBC*	LapB and LapC subunits homologs of the ABC transporter for LapA secretion in *P. fluorescens*. [1S]
PP0168	*lapA*	LapA surface-associated adhesion protein. [56, 1S]
PP0194	*algP*	AlgP alginate regulatory protein.
PP0216	*pp0216*	PAS/PAC sensor-containing GGDEF domain protein predicted to be a diguanylate cyclase.
PP0218	*pp0218*	Predicted signal transduction protein containing a membrane domain, an EAL and a GGDEF domain.
PP0337	*pp0337*	PAS/PAC and GAF sensor-containing GGDEF and EAL domain protein.
PP0369	*pp0369*	Predicted response regulator containing a CheY-like receiver domain and a GGDEF domain.
PPt04	*ppt04-pp0386*	*pp0386* encodes a predicted signal transduction protein containing a membrane domain, an EAL and a GGDEF domain.
PP0607	*pp0607*	Putative FimT type IV pili assembly protein.
PP0608	*pp0608-pp0609-pp0610-pilE-pp0612*	PilE type IV pili assembly protein.
PP0633	*pilCD-coaE-pp0630*	PilC and PilD type IV pili biogenesis proteins.
PP0634	*pilA*	PilA type IV pili prepilin.
PP0672	*morA*	*pp0672* codes for MorA a signal transduction protein containing a membrane domain, an EAL and a GGDEF domain.
PP0798	*pp0798*	GGDEF domain protein.
PP0803	*pp0803-pp0804-pp0805-lapF*	LapF seed colonization adhesion protein [36, 56].
PP0849	*ndk-pp0850-pilF-pp0852-ispG-hisS-pp0855-pp0856*	PilF type IV pili biogenesis protein/stability protein PilW.
PP0914	*bifA*	pp0914 codes for BifA a GGDEF and EAL domain protein with PDE activity [35].
PP1042	*xcpX*	XcpX type II secretion pathway protein. Related to QS in *P. aeruginosa* [2S].
PP1044	*uxpA-xcpPQR-S1-T1-UVWYZ-gspN*	XcpPQR-S1-T1-UVWYZ type II secretion pathway proteins. Related to QS in *P. aeruginosa* [2S].

PP1144	*pp1144*	Predicted signal transduction protein containing a membrane domain, an EAL and a GGDEF domain.
PP1155	*pp1155-pp1154*	YaiC/YhcK/ArdA family protein containing a GGDEF domain.
PP1218	*pp1218-tolQRAB-oprL-pp1224*	TolA biopolymer transport protein. Related to QS in *P. aeruginosa* [2S].
PP1280	*algIJFA*	AlgI alginate O-acetylation protein. Regulated by RoxSR QS enzymes [3S].
PP1371	*mcpG*	GABA-binding methyl-accepting chemotaxis transducer. Chemotaxis transducers are related to QS in *P. aeruginosa* [2S].
PP1383	*pp1383*	BenF-like porin homologue to PA0240 [4S].
PP1386	*ttgABC*	RND multidrug efflux system proteins, which in *P. aeruginosa* are related to QS [2S].
PP1408	*phaG*	Acyl-transferase possibly involved in rhamnolipid biosynthesis, which is related to biofilm structure [4S].
PP1411	*pp1411-pp1410*	*pp1411* encodes a predicted response regulator containing a CheY-like receiver domain and a GGDEF domain.
PP1427	*algU-mucA-algN*	AlgU RNA polymerase σ^{H} factor related to QS and adaptation to high temperatures [2S; 5S].
PP1450	*hlpBA*	HlpAB two-partner secretion system involved in root colonisation [6S].
PP1494	*pp1494*	*pp1494* codes for a WspR homolog response regulator containing a CheY-like receiver domain and a GGDEF domain.
PP1599	*pp1599-pp1600-lpxD*	LpxD UDP-3-O-[3-hydroxymyristoyl] glucosamine N-acyltransferase upregulated in biofilm [4S; 7S].
PP1623	*rpoS*	RNA polymerase σ factor RpoS.
PP1719	*pp1719-pp1718*	*pp1718* codes for a conserved domain protein that may contain an EAL domain.
PP1758	*pp1758-pp1759-pp1760-pp1761-pp1762*	*pp1761* encodes a sensory box protein/GGDEF family protein [may also contain EAL domain]
PP1890	*fimCD-pp1888*	Operon related to fimbrial biogenesis.
PP1891	*fimI*	FimI type 1 pili subunit.
PP2097	*pp2097*	Predicted signal transduction protein containing a membrane domain, an EAL and a GGDEF domain.
PP2357	*pp2357-pp2358-pp2359-pp2360-csuCDE*	Operon related to spore coat and fimbrial biogenesis.
PP2505	*pp2505*	GAF domain/GGDEF domain protein.
PP2557	*pp2557-pp2556-pp2555*	*pp2557* codes for a PAS/PAC sensor-box protein with GGDEF domain.
PP2629	*pp2629-pp2630.bcsFGRQABZC*	Cellulose biosynthesis operon.
PP3126	*pp3126*	Putative polysaccharide export protein.
PP3127	*pp3127-pp3128*	*pp3128* codes for a putative exopolysaccharide biosynthesis/transport protein.
PP3182	*pp3182-pp3281*	*pp3182* codes for predicted signal transduction protein containing a membrane domain, an EAL and a GGDEF domain.
PP3242	*pp3242*	Predicted response regulator containing a CheY-like receiver domain and a GGDEF domain.

PP3319	*pp3319*	Predicted signal transduction protein containing a membrane domain, an EAL and a GGDEF domain.
PP3396	*pp3396-pp3397-pp3398*	*pp3398* codes for a putative CsgB curli fiber surface-exposed nucleator.
PP3435	*pp3435-rarD-2*	*pp3435* codes for a predicted c-di-GMP phosphodiesterase class I containing an EAL domain.
PP3452	*pp3452*	Predicted response regulator containing a CheY-like receiver domain and a GGDEF domain.
PP3581	*pp3581*	Predicted signal transduction protein containing a membrane domain, an EAL and a GGDEF domain.
PP3672	*pp3672*	Predicted c-di-GMP phosphodiesterase class I with an EAL domain.
PP3711	*pp3711*	Predicted signal transduction protein containing a membrane domain, an EAL and a GGDEF domain.
PP3932	*pp3932*	Predicted c-di-GMP synthetase containing a GGDEF domain.
PP4004	*ftsK-lolA-pp4002-crcB-serS-cobA-2*	CrcB protein. In *P. aergionsa crc* mutants are biofilm deficient [4S].
PP4100	*pp4100-gacA-uvrC-pgsA*	*pp4099* codes for GacA DNA-binding response regulator involved in initial biofilm development in *P. aeruginosa* [4S].
PP4328	*pp4328-pp4329*	*pp4329* codes for a FlhB domain protein.
PP4340	*cheYZ*	CheY chemotaxis protein. Related to QS in *P. aeruginosa* [2S].
PP4344	*flhAF-fleN-fliA*	FlhA flagellar biosynthetic protein, FleN flagellar number regulator and FliA flagella σ^F factor.
PP4361	*fliK-pp4360-fliLMNOPQR-flhB*	Operon related to flagella biosynthesis.
PP4364	*pp4364-pp4363-pp4362*	*pp4364* encodes a putative anti-σ^F factor antagonist.
PP4367	*fliHIJ*	Operon related to flagella biosynthesis.
PP4370	*fliEFG*	Operon related to flagella biosynthesis.
PP4372	*fleSR*	FleS and FleR flagella two-component regulatory system.
PP4373	*fleQ*	FleQ transcriptional regulator.
PP4375	*fliST*	FliS flagellar biosynthetis protein.
PP4376	*fliD*	FliD flagellar cap protein.
PP4378	*fliC-fleL*	FliC flagellin. Related to biofilm growth and survival in low-water content habitats [8S].
PP4386	*flgFGHIJKL*	Operon related to flagella biosynthesis.
PP4391	*flgBCDE-pp4387*	Operon related to flagella biosynthesis.
PP4393	*cheV3-R*	CheV3 and CheR chemotaxis proteins.
PP4394	*flgA*	FlgA flagella basal body P-ring formation protein.
PP4395	*flgMN*	FlgM negative regulator of flagellin synthesis.
PP4405	*pp4405*	Predicted PAS/PAC sensor-containing a GGDEF domain.
PP4470	*algZ*	AlgZ alginate biosynthesis transcriptional activator.
PP4519	*pp4519*	Putative LapE, LapA secretion outer membrane protein.
PP4615	*pp4615*	Seed colonization protein DdcA [9S].
PP4641	*pp4641*	CstA carbon starvation protein. Related to biofilm. attachment [56].

PP4671	*pp4671-pp4670*	*pp4670* codes for a GGDEF domain protein.
PP4693	*dksA-pp4694*	DksA is a stringent response regulator involved in biofilm dispersal [48].
PP4695	*cbrB*	CbrAB two-component system.
PP4944	*pp4944-pp4943-pp4942-pp4941-pp4940-pp4939-pp4938*	*pp4943* codes for a *wapH* homolog, core lipopolysaccharide biosynthesis, related to EPS production [10S].
PP4959	*pp4959-pp4958-pp4957*	*pp4959* codes for a predicted signal transduction protein containing a membrane domain, an EAL and a GGDEF domain.
PP4992	*pilGHIJ-pp4988-pp4987*	Operon related to pili biosynthesis.
PP4995	*algH-pp4996-pyrRBC*	AlgH alginate biosynthesis nucleoside diphosphate kinase regulator.
PP5083	*pilMN-pp5081-pilQ-aroK*	Operon related to pili biosynthesis.
PP5093	*pilT*	PilT type IV pili twitching motility protein. Related to QS in *P. aeruginosa* [2S].
PP5180	*potF-1GHI*	Putrescine ABC transporter, periplasmic putrescine-binding protein downregulated in biofim [7S].
PP5181	*potF-2*	Putrescine ABC transporter, periplasmic putrescine-binding protein downregulated in biofim [7S].
PP5263	*pp5263*	Predicted signal transduction protein containing a membrane domain, an EAL and a GGDEF domain.
