# Supplementary material for: Complex Interplay between FleQ, Cyclic Diguanylate and Multiple σ Factors Coordinately Regulates Flagellar Motility and Biofilm Development in Pseudomonas putida
Source: PLoS One. 2016 Sep 16;11(9):e0163142. doi: 10.1371/journal.pone.0163142 (PMC5026340; doi:10.1371/journal.pone.0163142)
Supplement: S2 Table — The results shown are the averages and standard deviations of at least 3 independent experiments. (PDF) [file pone.0163142.s007.pdf]

S2 Table. Results of the screenings for FleQ- and c-di-GMP-dependent regulation. The results shown are the averages and standard deviations of at least 3 independent experiments.

	FleQ screening					c-di-GMP screening				
	Wild-type		*fleQ*⁻		Ratio	High c-di-GMP		Low c-di-GMP		Ratio low/high
ORF ID	Average	SD	Average	SD	wild-type/*fleQ*⁻	Average	SD	Average	SD	c-di-GMP
**PP0127**	63	36	58	29	1.1	30	11	36	17	1.3
**PP0131**	43	20	50	35	0.9	24	9	23	6	0.9
**PP0133**	97	44	97	51	1	49	14	53	11	1.1
**PP0164**	186	83	148	79	1.3	71	17	97	8	1.4
**PP0167**	78	40	57	32	1.4	51	11	35	8	0.7
**PP0168**	149	70	53	31	2.8	82	28	51	5	0.6
**PP0194**	50	27	40	25	1.2	32	15	28	8	0.9
**PP0216**	54	24	49	28	1.1	28	9	26	10	0.9
**PP0218**	269	161	345	176	0.8	146	84	142	61	1.0
**PP0337**	259	118	241	120	1.1	108	18	151	19	1.4
**PP0369**	66	41	50	30	1.3	36	6	27	8	0.8
**PPt04**	125	56	77	45	1.6	46	18	64	13	1.4
**PP0607**	49	21	48	35	1	26	6	24	4	0.9
**PP0608**	49	27	57	38	0.9	30	16	31	12	1.0
**PP0633**	125	61	128	68	1	28	8	34	6	1.3
**PP0634**	80	42	80	46	1	59	14	65	19	1.1
**PP0672**	242	111	151	82	1.6	90	17	159	27	1.7
**PP0798**	125	61	147	75	0.9	54	13	72	10	1.3
**PP0803**	347	428	188	130	1.9	21	9	44	16	2.0
**PP0849**	333	162	313	147	1.1	146	35	172	28	1.1
**PP0914**	159	71	117	71	1.4	58	29	128	24	2.0
**PP1042**	58	33	52	32	1.1	31	12	32	8	1.0
**PP1044**	43	27	41	26	1	32	14	24	6	0.8
**PP1144**	118	37	77	43	1.5	70	76	72	42	1.0
**PP1155**	129	54	130	71	1	47	19	55	7	1.1

**PP1218**	38	20	35	19	1.1	18	9	18	7	1.0
**PP1280**	45	24	41	25	1.1	21	9	20	9	0.9
**PP1371**	75	47	26	19	2.9	31	15	40	8	1.3
**PP1383**	37	19	39	23	0.9	22	7	17	6	0.8
**PP1386**	158	100	173	68	0.9	66	22	66	19	1.0
**PP1408**	72	60	68	38	1.1	27	8	24	10	0.9
**PP1411**	67	39	59	34	1.1	35	13	33	8	0.9
**PP1427**	437	202	493	274	0.9	201	116	222	54	1.1
**PP1450**	54	31	53	31	1	33	14	31	16	0.9
**PP1494**	53	33	46	30	1.2	29	10	29	13	1.0
**PP1599**	43	20	34	23	1.3	20	10	29	22	1.4
**PP1623**	54	37	54	28	1	25	10	32	20	1.3
**PP1719**	571	274	626	340	0.9	249	32	281	36	1.1
**PP1758**	262	116	289	148	0.9	112	12	167	49	1.4
**PP1890**	35	20	32	21	1.1	19	9	20	11	1.0
**PP1891**	34	20	33	20	1	21	11	18	7	0.8
**PP2097**	132	66	95	52	1.4	73	36	83	19	1.1
**PP2357**	211	99	159	87	1.3	52	17	98	7	2.0
**PP2505**	48	28	38	29	1.3	27	13	23	4	0.8
**PP2557**	220	110	247	119	0.9	102	17	114	10	1.1
**PP2629**	863	398	308	138	2.8	728	114	418	34	0.6
**PP3126**	155	76	175	113	0.9	196	178	74	15	0.4
**PP3127**	48	28	45	28	1.1	25	8	24	6	1.0
**PP3182**	49	28	51	28	1	31	15	23	4	0.7
**PP3242**	232	186	287	214	0.8	102	87	113	52	1.1
**PP3319**	441	191	590	336	0.7	72	13	139	26	2.0
**PP3396**	180	79	196	103	0.9	79	13	103	7	1.3
**PP3435**	138	67	140	83	1	57	9	71	10	1.3
**PP3452**	88	44	94	48	0.9	47	17	45	7	1.0
**PP3581**	256	116	319	153	0.8	122	22	155	8	1.3
**PP3672**	35	21	33	22	1.1	23	11	19	9	0.8
**PP3711**	163	104	72	39	2.3	63	15	69	7	1.1
**PP3932**	157	68	272	138	0.6	123	17	78	41	0.6

**PP4004**	143	68	146	81	1	69	11	79	10	1.1
**PP4100**	285	177	185	123	1.5	112	58	180	83	1.7
**PP4328**	305	146	153	86	2	81	16	181	20	2.5
**PP4340**	53	33	57	33	0.9	32	16	30	9	1.0
**PP4344**	72	33	26	18	2.7	34	13	46	7	1.4
**PP4361**	47	26	39	23	1.2	23	9	23	5	1.0
**PP4364**	465	226	608	324	0.8	158	33	216	30	1.4
**PP4367**	59	32	61	36	1	32	16	31	7	1.0
**PP4370**	38	22	34	22	1.1	24	12	18	8	0.8
**PP4372**	37	20	30	21	1.2	21	13	17	7	0.8
**PP4373**	55	27	55	36	1	33	18	30	15	0.9
**PP4375**	189	90	48	32	3.9	52	29	134	32	2.5
**PP4376**	52	28	47	25	1.1	30	11	32	9	1.1
**PP4378**	148	61	54	28	2.8	155	28	1219	152	10.0
**PP4386**	38	20	37	23	1	26	12	21	9	0.8
**PP4391**	315	142	35	20	9.1	63	17	233	35	3.3
**PP4393**	453	373	156	84	2.9	106	61	216	30	2.0
**PP4394**	222	139	64	36	3.5	59	13	110	40	2.0
**PP4395**	863	507	333	166	2.6	166	55	494	113	3.3
**PP4405**	128	94	94	46	1.4	49	18	55	15	1.1
**PP4470**	306	263	201	123	1.5	91	22	125	26	1.4
**PP4519**	605	397	541	651	1.1	388	130	381	166	1.0
**PP4615**	192	138	114	70	1.7	70	18	49	9	0.7
**PP4641**	423	157	509	357	0.8	151	35	204	17	1.4
**PP4671**	35	17	35	20	1	24	14	21	5	0.8
**PP4693**	140	66	146	73	1	64	17	77	11	1.3
**PP4695**	283	131	326	156	0.9	132	19	181	54	1.4
**PP4944**	464	146	489	234	0.9	230	66	238	15	1.0
**PP4959**	290	229	358	232	0.8	57	50	65	40	1.1
**PP4992**	536	225	516	244	1	198	18	297	61	1.4
**PP4995**	326	151	225	159	1.5	158	23	159	20	1.0
**PP5083**	57	31	54	34	1.1	24	9	25	6	1.1
**PP5093**	62	35	59	37	1.1	33	8	29	5	0.9

**PP5180**	56	28	61	33	0.9	34	10	32	10	0.9
**PP5181**	127	58	141	76	0.9	61	17	64	10	1.0
**PP5263**	34	18	33	21	1	24	12	18	6	0.8
**pMRB1**	56	7	51	2	1.1	22	10	21	7	0.9
**KT2442**	51	23	54	20	1	26	4	30	11	1.1
