# Supplementary material for: Complex Interplay between FleQ, Cyclic Diguanylate and Multiple σ Factors Coordinately Regulates Flagellar Motility and Biofilm Development in Pseudomonas putida
Source: PLoS One. 2016 Sep 16;11(9):e0163142. doi: 10.1371/journal.pone.0163142 (PMC5026340; doi:10.1371/journal.pone.0163142)
Supplement: S3 Table — Prediction was performed according to [18]. 1Coordinates are relative to the start codons. 2A plus (+) sign designates the strand corresponding to the lapA or bcs transcript 3Positions matching the consensus are indicated in bold. (PDF) [file pone.0163142.s008.pdf]

S3 Table. Predicted FleQ binding sites at the P*lapA* and P*bcsD* promoter regions.

Prediction was performed according to [18] [1]Coordinates are relative to the start codons. [2]A plus (+) sign designates the strand corresponding to the *lapA* or *bcs* transcript [3]Positions matching the consensus are indicated in bold.

Promoter	Start[1]	End[1]	Strand[2]	P-value	Sequence[3]
P*lapA*	-153	-140	+	8.36×10^{-6}	**GTCAATA**GT**TTG**G**C**
P*lapA*	-114	-101	+	1.58×10^{-5}	**G**A**C**G**G**A**A**T**ATTGAC**
P*lapA*	-668	-655	+	3.12×10^{-5}	**GTCAGTAAA**CA**G**G**C**
P*bcsD*	-147	-134	+	2.65×10^{-5}	**GTC**G**TT**TTT**TTGAC**
P*bcsD*	-107	-94	+	9.44×10^{-5}	**G**CTG**T**AT**AATTGAC**
**Consensus**					**GTCanTAAAtTGAC**
